# *Leishmania infantum* Modulates Host Macrophage Mitochondrial Metabolism by Hijacking the SIRT1-AMPK Axis

**DOI:** 10.1371/journal.ppat.1004684

**Published:** 2015-03-04

**Authors:** Diana Moreira, Vasco Rodrigues, Maria Abengozar, Luis Rivas, Eduardo Rial, Mireille Laforge, Xiaoling Li, Marc Foretz, Benoit Viollet, Jérôme Estaquier, Anabela Cordeiro da Silva, Ricardo Silvestre

**Affiliations:** 1 Parasite Disease Group, IBMC—Instituto de Biologia Molecular e Celular, Universidade do Porto, Porto, Portugal; 2 Instituto de Investigação e Inovação em Saúde, Universidade do Porto, Porto, Portugal; 3 CNRS FRE 3636, Université Paris Descartes, Paris, France; 4 Unidad Metabolómica, Interacciones y Bioanálisis (UMIB), Centro de Investigaciones Biológicas (CSIC), Madrid, Spain; 5 Department of Cellular and Molecular Medicine, Centro de Investigaciones Biológicas (CSIC), Madrid, Spain; 6 Laboratory of Signal Transduction, National Institute of Environmental Health Sciences, Research Triangle Park, North Carolina, United States of America; 7 INSERM U1016, Institut Cochin, Paris, France; 8 CNRS UMR 8104, Paris, France; 9 Université Paris Descartes, Sorbonne Paris Cité, Paris, France; 10 Université Laval, Centre de Recherche du CHU de Québec, Québec, Canada; 11 Departamento de Ciências Biológicas, Faculdade de Farmácia, Universidade do Porto, Porto, Portugal; McGill University, CANADA

## Abstract

Metabolic manipulation of host cells by intracellular pathogens is currently recognized to play an important role in the pathology of infection. Nevertheless, little information is available regarding mitochondrial energy metabolism in *Leishmania* infected macrophages. Here, we demonstrate that during *L. infantum* infection, macrophages switch from an early glycolytic metabolism to an oxidative phosphorylation, and this metabolic deviation requires SIRT1 and LKB1/AMPK. SIRT1 or LBK1 deficient macrophages infected with *L. infantum* failed to activate AMPK and up-regulate its targets such as *Slc2a4* and *Ppargc1a*, which are essential for parasite growth. As a result, impairment of metabolic switch caused by SIRT1 or AMPK deficiency reduces parasite load *in vitro* and *in vivo*. Overall, our work demonstrates the importance of SIRT1 and AMPK energetic sensors for parasite intracellular survival and proliferation, highlighting the modulation of these proteins as potential therapeutic targets for the treatment of leishmaniasis.

## Author Summary

*Leishmania infantum*, a causative agent of visceral leishmaniasis, is able to infect host macrophages and modulate a myriad of signalling pathways that contributes to the disease outcome. In order to survive, *L. infantum* must compete with the host for the same metabolic resources, however scarce attention has been dedicated to clarify the potential interference of the parasite with the host metabolic pathways and its impact for the infection outcome. We analysed the macrophage metabolic alterations induced by *L. infantum* focusing on host energetic players exploited by the parasite. We describe that *L. infantum* induced a metabolic switch from an early aerobic glycolytic environment to a later mitochondrial metabolism. In this process, *L. infantum* modulates important energetic sensors of the host, such as the SIRT1-LKB1-AMPK axis. This triad is important for the recovery of the host energetic status and also for the parasite survival. With this work, we demonstrate that the host SIRT1-LKB1-AMPK axis has a crucial impact on the parasite survival *in vitro* and *in vivo*.

## Introduction

Visceral leishmaniasis (VL) is a potentially fatal vector-borne disease caused by protozoan *Leishmania donovani* and *L. infantum* parasites. Infection of the mammalian host is initiated with the inoculation of the flagellated promastigote forms during the sand fly bloodmeal. Once inside the host, *Leishmania* parasites are phagocyted mainly by macrophages, where they reside inside the phagolysosomal compartment and differentiate into obligate intracellular amastigotes. The interplay between parasite factors and host responses is crucial for the final outcome of infection, thereby for disease pathogenesis [1].

Extensive studies have focused on the characterization of *Leishmania* virulence factors and the strategies developed by the parasite to manipulate host intracellular signaling pathways towards immune evasion and survival [2,3]. Yet, scarce attention has been paid to the manipulation of host nutrient and energy sources by *Leishmania* parasites despite the competition of both organisms for identical resources. Mitochondrion plays a crucial role during apoptotic cell death [4], is the site of ATP synthesis and is where essential metabolic pathways take place. These include the citric acid cycle, fatty acid oxidation, the synthesis and degradation of amino acids and the synthesis of iron–sulfur clusters and heme. Mitochondria are dynamic compartments that rearrange in response to stress and changes in nutrient availability or oxygen concentration. Metabolic reprogramming of cells is an integral view of cell metabolism in order to satisfy cell proliferation and survival requirements. Despite its recognized importance in disease pathogenesis, there is limited understanding of various aspects of mitochondrial bioenergetics in the context of host-pathogen interactions. From a bioenergetic and metabolic perspective, intracellular pathogens may benefit from existing resources and can manipulate the host for their own profit to fulfill their requirements. The intracellular growth of *Trypanosoma cruzi* was recently shown to rely predominantly on the energy production, nucleotide metabolism and fatty acid oxidation of the host [5]. Although the metabolic manipulation of host cells is recognized to play an important role in the pathologic processes of infection [6], the puzzle is even more complex when the invading pathogen shares many metabolic pathways with the host, a frequent case for protozoan infections.

AMP-activated protein kinase (AMPK), a central cellular signaling hub involved in the regulation of energy homeostasis, has been suggested as a potential attractive target for pathogen manipulation [6]. As a paradigm, hijacking of AMPK pathway by viruses has been documented [7], with its activation or inhibition being strictly dependent on the species involved but invariably satisfying viral interests. From a metabolic point of view, AMPK protein complex is activated upon changes in the AMP/ATP ratio mirroring shortage of nutrients [8]. In addition to allosteric regulation by AMP, AMPK activation is controlled by phosphorylation on conserved threonine 172 (Thr172) residue namely by two upstream kinases, the tumor suppressor kinase LKB1 (liver kinase B1) [9,10] or the calcium/calmodulin-dependent protein kinase kinases, whose activity is dependent on Ca^2+^ levels [11,12]. As a final output, AMPK activation tips the energetic balance by switching on catabolic pathways. Akin, sirtuin proteins deacetylate key targets in response to intracellular NAD^+^ levels fluctuations, playing a role as cellular energetic sensors [13]. In particular, the partnership between SIRT1 and AMPK in mediating the cellular response to nutrient availability has been described [14]. Nevertheless, the promiscuous relation of AMPK and SIRT1 appears to be cell specific and their putative role during infection remains elusive.

In this work, we dissected the macrophage metabolic pathways engaged by *Leishmania* parasites and elucidated the consequences of this metabolic hijacking for parasite growth and persistence. Bioenergetic flux analysis of *L. infantum* infected macrophages revealed a two-step infection process, including an initial transient aerobic glycolytic phase followed by a metabolic shift towards mitochondrial metabolism. This metabolic switch requires the catalytic activities of SIRT1 and LKB1 as well as the downstream AMPK energetic sensor. While allowing the metabolic recovery of the host cell, the activation of the SIRT1/AMPK axis ultimately contributes to parasite survival *in vitro* and *in vivo*. Our findings point out that the macrophage metabolism could be a potential therapeutic target against leishmaniasis diseases.

## Results

### Bioenergetic profile switch during *L. infantum*-infected macrophages

**Fig 1:**
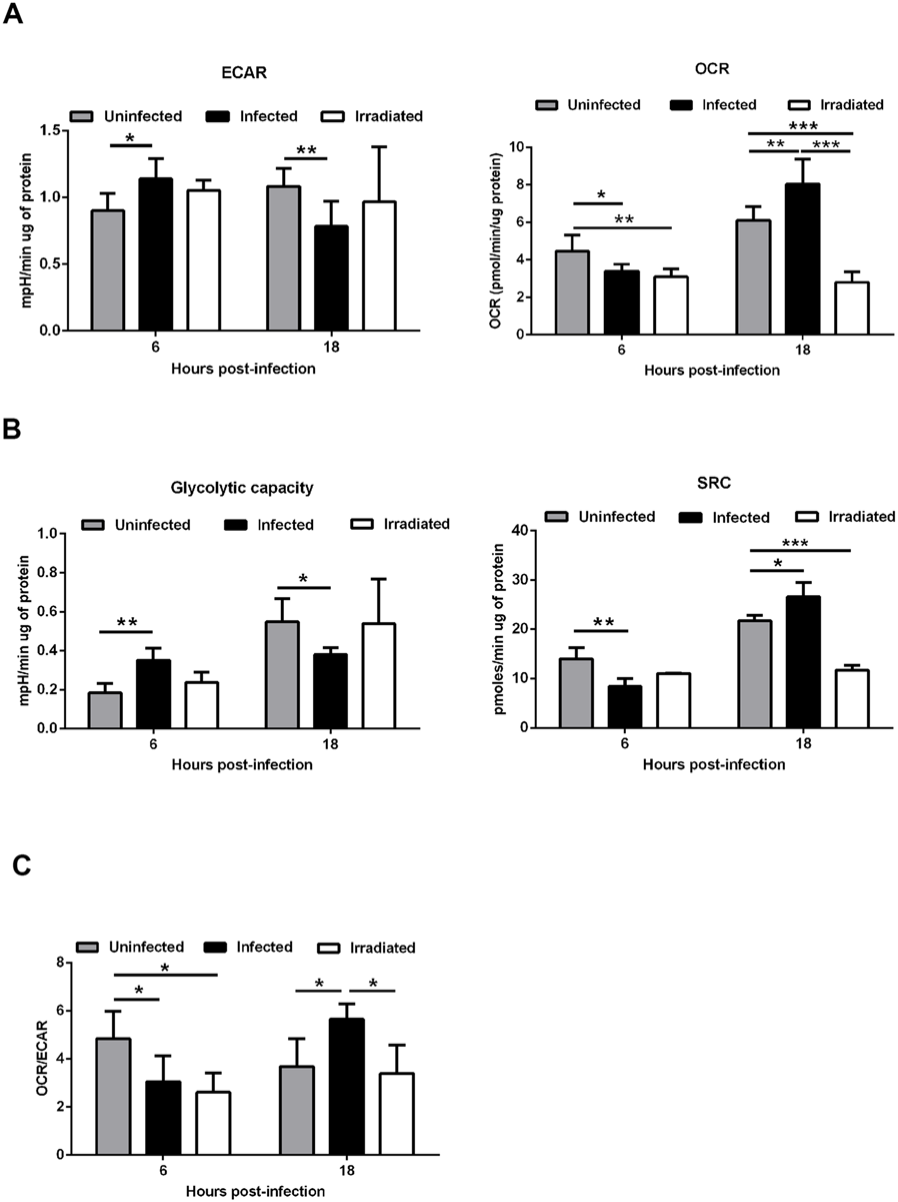
*Leishmania infantum* modulates host macrophages bioenergetic profile. BMMo were infected with live or irradiated *L. infantum* (1:10 ratio) for 6 and 18 hours. At each time point ECAR and OCR was measured in real time under basal conditions and in response to specific inhibitors. (A) Means ± SD of basal OCR and ECAR and (B) SRC and glycolytic capacity obtained from eight independent experiments. The bioenergetics profile of ECAR (S1A Fig.) and OCR (S1B Fig.) was traced for the referred time points. (C) The respective OCR/ECAR ratio was calculated. Viability (S2A-B Fig.), infection profile (S2C Fig.) and growth (S2D Fig.) of irradiated *L. infantum* were determined. *p <0.05, **p <0.01.

To address how *Leishmania infantum* infection impacts on macrophage metabolism and bioenergetic state, we quantified the extracellular acidification rate (ECAR), a consequence of lactate production, and the mitochondrial oxygen consumption rate (OCR), to monitor the rate of oxidative phosphorylation, using live cell extracellular flux analysis. Six hours post-infection, higher basal ECAR levels were observed in infected bone marrow derived macrophages (BMMo) compared to uninfected cells (Fig. 1A and S1A Fig.), corresponding to a higher glycolytic capacity (Fig. 1B and S1A Fig.). At the same time point, a significant reduction of the OCR values was observed on infected BMMo (Fig. 1A and S1B Fig.). Accordingly, the spare respiratory capacity (SRC), as calculated by the difference between the maximal OCR determined in the presence of the uncoupler FCCP and basal value was significantly reduced on infected BMMo indicating lower levels of mitochondrial respiration (Fig. 1B and S1B Fig.). In contrast, this pattern was altered after 18 hours post-infection (p.i.), where higher respiration levels were observed in infected BMMo as evidenced by increased SRC (Fig. 1B and S1B Fig.). Concomitantly to the increase in OCR values, ECAR was reduced at 18 hours p.i. in infected BMMo (Fig. 1A and S1A Fig.). These variations were reflected by a significant variation on the OCR/ECAR ratio (Fig. 1C). To understand if live intracellular parasites by themselves contribute to increase the glycolytic metabolism, BMMo were challenged with irradiated *L. infantum* promastigotes. Irradiated promastigotes maintain membrane integrity for 6 hours as shown by 7-Aminoactinomycin D (7-AAD) staining (S2A-B Fig.) and were phagocyted in a similar manner as live parasites (S2C Fig.). Intra or extracellularly, the irradiated promastigotes were unable to grow; in fact a dramatic increase in membrane permeabilization associated with cell death was observed when measured 18 hours post-irradiation (S2A-D Fig.). Our results showed no difference in the ECAR parameter between uninfected and irradiated *L. infantum* BMMo (Fig. 1A-C and S1A-B Fig.), while the levels of OCR and SRC were always lower in the latter, yet without affecting the OCR/ECAR ratio at 18 hours p.i. These results indicate that the observed phenotype is mostly due to the manipulation of the host by parasite and not caused by the presence of the parasite.

Altogether, our results demonstrated that infection of BMMo with live *L. infantum* is associated with a transient bioenergetic profile towards aerobic glycolysis with a concomitant reduction of mitochondria function early after infection followed by a metabolic shift towards mitochondrial metabolism.

### Early glycolysis is associated with increased levels of key glycolytic enzymes in *L. infantum*-infected macrophages

**Fig 2:**
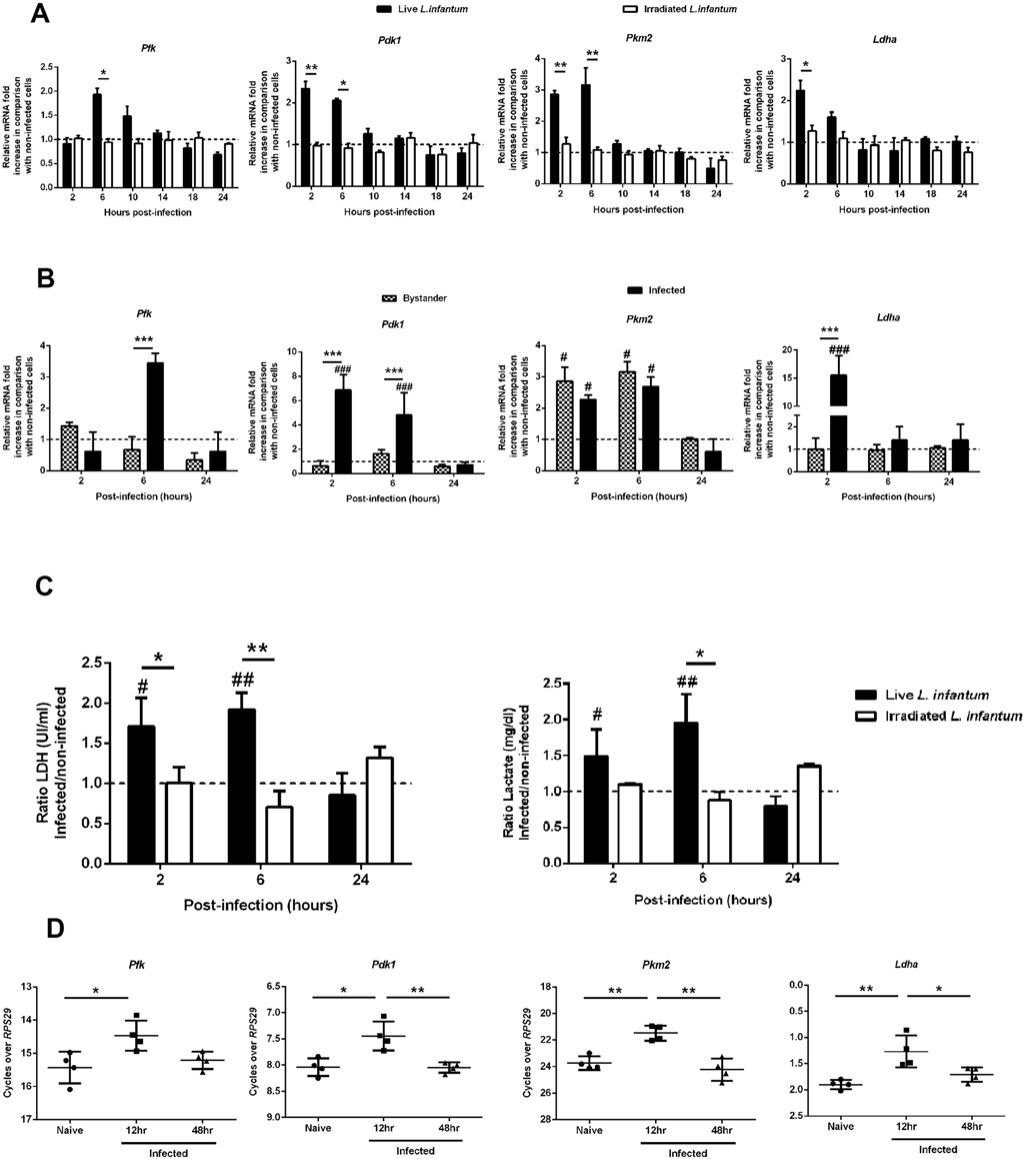
*L. infantum* alters host glycolytic transcription profile. (A) BMMos were infected with live or irradiated *L. infantum* (1:10 ratio). The transcription levels of the glycolytic genes *Pfk*, *Pdfk1*, *Pkm2* and *Ldha* were analyzed by qPCR. Additional glycolytic genes were evaluated by qPCR (S3B Fig.). All the enzymes are depicted in a representative scheme in S3A Fig. (B) Bystander and infected BMMos were sorted after 2, 6 or 24 hours post-infection and the transcript levels of *Pfk*, *Pdfk1*, *Pkm2* and *Ldha* analyzed. (C) Bars represent the mean ratios ± SD of the LDH activity and lactate secretion between live or irradiated *L. infantum* infected and uninfected BMMo. (D) The transcription levels of all the referred glycolytic genes in naïve and infected splenic macrophages were analyzed at 12 and 48 hours post-infection (S3C Fig.). The transcription levels of the glycolytic genes *Pfk*, *Pdk1*, *Pkm2* and *Ldha* (S3D Fig.) as well as LDH activity and lactate secretion (S3E Fig.) were analyzed in BMMo infected with axenic *L. infantum* amastigotes. Means ± SD are from three independent experiments. *p <0.05, **p <0.01, ***p <0.001. Significant differences between infected or bystander and uninfected BMMo ^#^p <0.05, ^###^p <0.001.

Glycolysis, despite its lower energetic efficiency as compared with to oxidative phosphorylation, is far quicker than oxidation of pyruvate in the mitochondria. Pyruvate derived from glycolysis is either reduced to lactate or enters into the tricarboxylic acid (TCA) cycle in processes involving several kinases. We assessed the expression of hexokinase 1 (*Hk1*), hexokinase 2 (*Hk2*), pyruvate kinase M1 (*Pkm1*), phosphofructokinase (*Pfk*), pyruvate dehydrogenase kinase 1 (*Pdk1*), pyruvate kinase M2 (*Pkm2*) and lactate dehydrogenase a (*Ldha*) transcripts (S3A Fig.). The transcripts were analyzed in BMMo challenged with live or irradiated *L. infantum* to ascertain the mechanisms responsible for the bioenergetic profile observed. In the first hours of infection (2 and 6 hours), live parasites induce a significant increase of *Pfk*, *Pdk1*, *Pkm2* and *Ldha* transcripts as compared to either uninfected cells or challenged with irradiated promastigotes (Fig. 2A). All up-regulated genes returned to their initial levels approximately at 10–14h p.i. No differences were found in the transcripts of *Hk1*, *Hk2* and *Pkm1* glycolytic enzymes (S3B Fig.). Further experiments performed in sorted infected and bystander BMMo demonstrated that the increase of *Pfk*, *Pdk1* and *Ldha* transcripts was specific to infected cells (Fig. 2B), with the exception of *Pkm2* that is equally transcribed in both populations. Importantly, the raise of *Ldha* transcript paralleled the increase of LDH enzymatic activity and concomitant lactate secretion (Fig. 2C). The LDH enzymatic activity or lactate secretion was not due to the presence of intracellular parasites since only live but not irradiated promastigotes displayed the referred phenotype (Fig. 2C). In order to determine whether the *in vitro* profile observed is reminiscent to *in vivo* situation, macrophages sorted from the spleens of mice infected with CFSE-labelled *L. infantum* were analyzed. As before, an increase of glycolytic genes was observed early after infection (Fig. 2D and S3C). In order to evaluate the impact of the differentiation process from promastigotes to amastigotes, we infected BMMo with axenic amastigotes. Unlike promastigote-challenged macrophages, infection with axenic amastigotes was not associated with increased LDH activity or lactate secretion (S3E Fig.). In agreement, amastigote infection failed to upregulate the mRNA levels of glycolytic enzymes as compared with uninfected macrophages, with the exception of *Pdk1*, a regulator of the activity of the pyruvate dehydrogenase complex (S3D Fig.). Altogether, these results demonstrated that the burst of aerobic glycolysis is associated with early upregulated expression of key glycolytic kinases.

### Switch to oxidative phosphorylation in *L. infantum*-infected macrophages is associated with increased expression of PGC-1α

**Fig 3:**
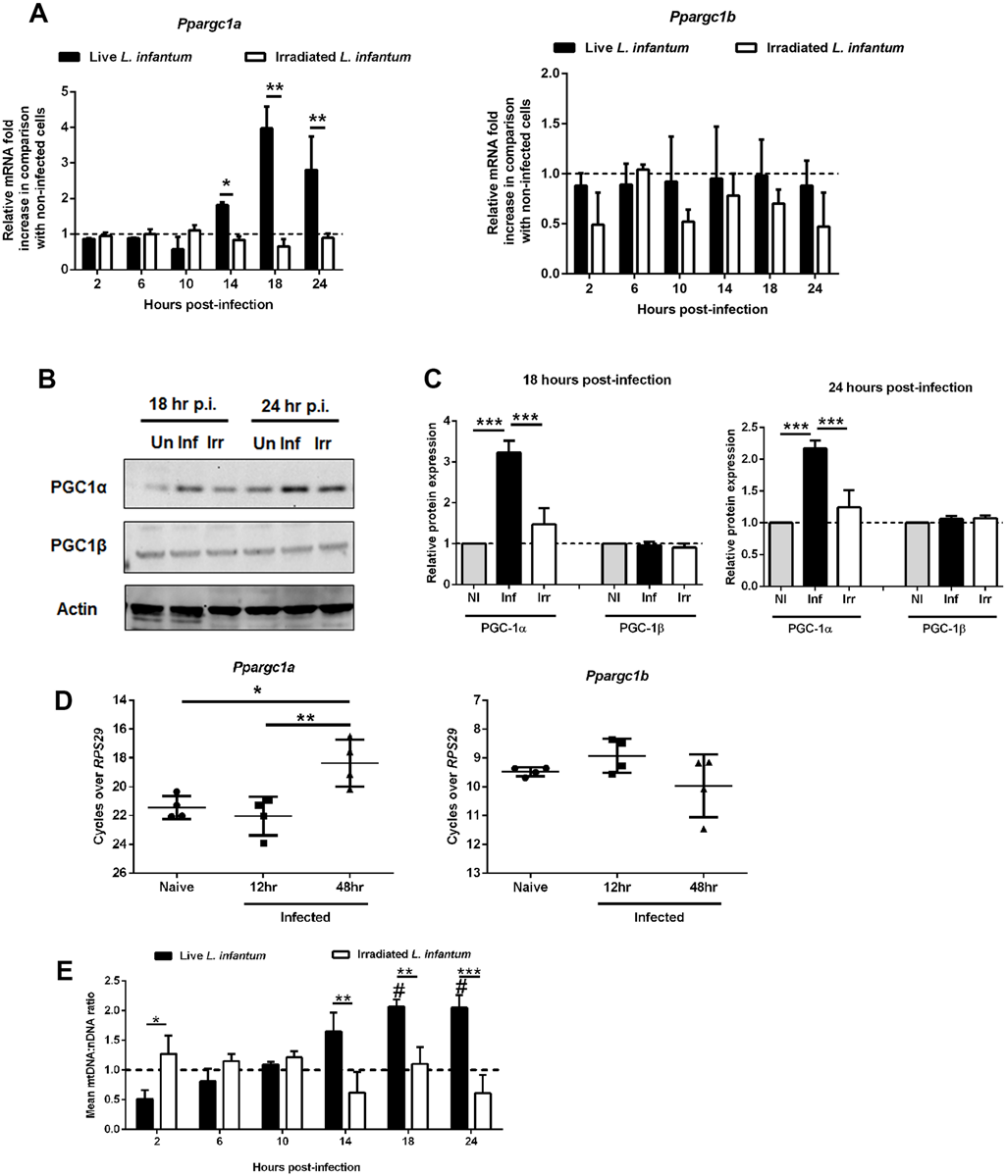
Alteration of host mitochondrial biogenesis by *L. infantum* infection. (A) BMMos were infected with live and irradiated *L. infantum* (1:10 ratio). At different time points the transcript levels of *Ppargc1a* and *Ppargc1b* were analyzed by qPCR. (B) Total protein extracts were prepared from uninfected (Un) or BMMo cultured with live (Inf) or irradiated (Irr) parasites. Immunoblots were probed with specific antibodies for PGC-1α and PGC-1β. Actin was used as loading control. (C) Quantification of both proteins is expressed as folds in comparison to uninfected BMMo after normalization with actin. (D) The transcript levels of *Ppargc1a* and *Ppargc1b* was performed in naïve and infected macrophages recovered from the spleen of Balb/c mice after 12 and 48 hours of infection. (E) Mitochondrial DNA/nuclear DNA ratio was determined at different times of infection and the transcription of nuclear genes encoding for mitochondrial complexes both *in vitro* (S4A-B Fig.) and *ex vivo* (S4C Fig.) were analyzed. The *Ppargc1*a transcript (S4D Fig.) and the mtDNA/nDNA ratio (S4E Fig.) were determined in BMMo infected with axenic amastigotes. Means ± SD are from four independent experiments. #p <0.05 in comparison to uninfected cells. *p <0.05, **p <0.01, ***p <0.001. Significant differences between infected and uninfected BMMo ^#^p <0.05.

To gain insight into the regulation of mitochondrial adaptations during the late phase of macrophage infection by *L. infantum*, we analyzed the peroxisome proliferator-activated receptors (PPARs), in particular the levels of PPAR-γ coactivator-1α (PGC-1α) and PPAR-γ coactivator-1β (PGC-1β), known to induce oxidative metabolism and mitochondrial biogenesis [15]. BMMo infected with live, but not with irradiated parasites, showed increased levels of *Ppargc1a*, but not *Ppargc1b*, transcripts peaking at 18 hours (Fig. 3A). Consistent with the transcriptional analysis, only BMMo infected with viable parasites presented higher PGC-1α protein levels at 18 and 24 hours (Fig. 3B). The increase of *Ppargc1a* transcripts was also observed in splenic macrophages recovered from mice at 48 hours post-infection (Fig. 3D). Accompanying the increase PGC-1α levels, we detected increased mitochondrial biogenesis in infected BMMo, as shown by an increment of mitochondrial DNA/nuclear DNA ratio upon 14 hours post-infection (Fig. 3E), and of nuclear genes encoding for mitochondrial complexes namely, *Ndufa9* (complex I) and *Cox4* (complex IV) both in *in vitro* infected BMMo or macrophages recovered from the spleen of infected mice (S4A-S4C Fig.). As found with promastigotes, amastigotes infected cells displayed an increase of the transcription levels of *Ppargc1a* (S4D Fig.), and higher levels of mitochondrial DNA/nuclear DNA ratio (S4E Fig.).

In conclusion, the metabolic regimen towards oxidative phosphorylation is a common mechanism for both *L. infantum* forms.

### AMPK activation in *L. infantum*-infected macrophages

**Fig 4:**
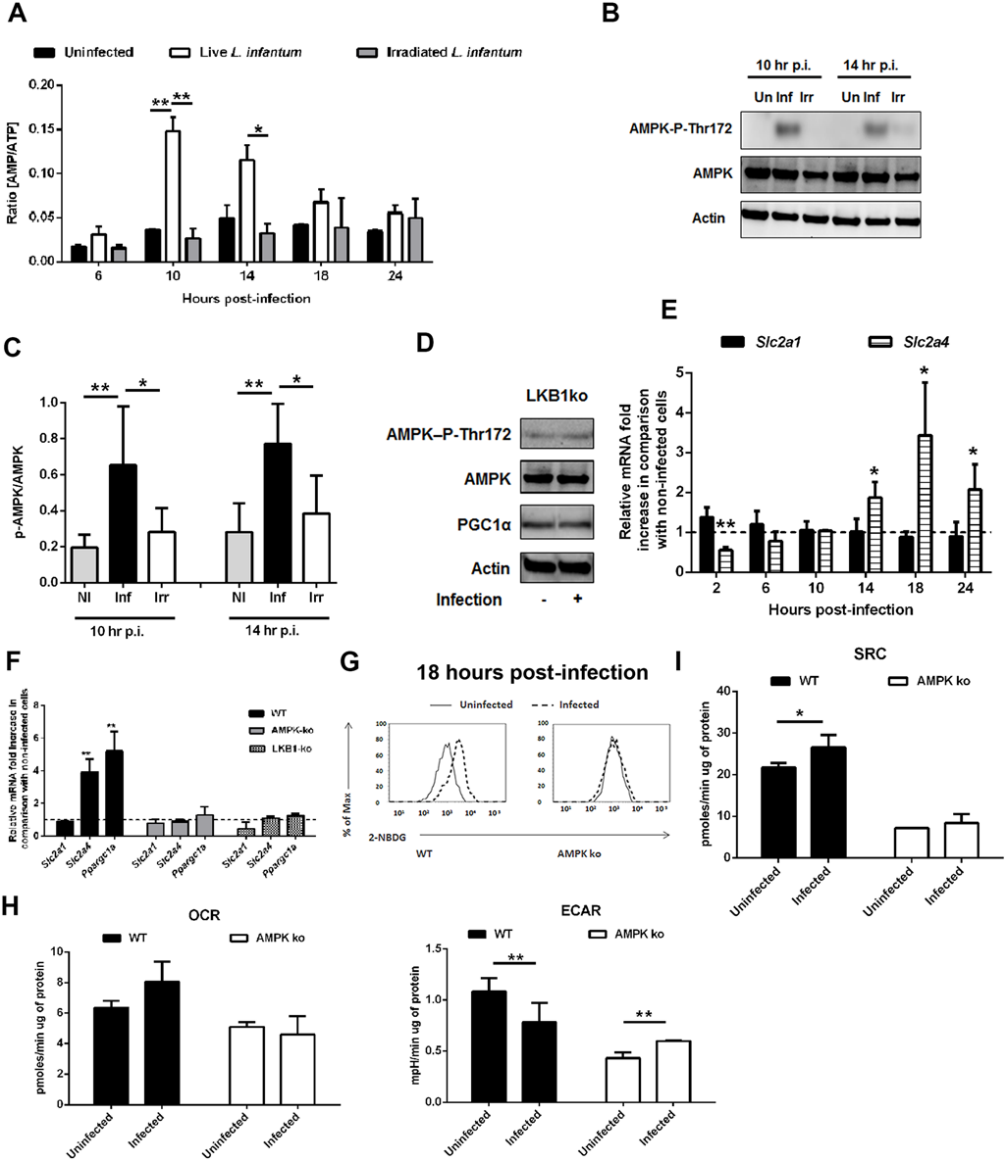
*L. infantum* activates host AMPK signaling for the recovery of mitochondrial functions. (A) At defined time points post-infection (p.i.), total ATP and AMP values were determined on BMMo infected with live or irradiated *L. infantum* (1:10 ratio). The AMP/ATP ratio is shown. AMP (S5A Fig.) and ATP (S5B Fig.) absolute levels were determined. Total ATP levels were analyzed in cells infected at different parasite doses (S5C Fig.), in sorted infected and bystander cells (S5D Fig.) and in cells infected with axenic amastigotes (S5E Fig.). (B) Immunoblots were probed with specific antibodies for AMPK-P-Thr172 and AMPK using actin as loading control in cells infected with promastigotes and amastigotes (S5F Fig.). (C) Quantification of both proteins is expressed as folds in comparison to uninfected BMMo after normalization with actin. (D) Uninfected and *L. infantum* infected BMMo from LKB1KO mice were analyzed at 14 hours p.i. for the expression levels of AMPK-P-Thr172, AMPK and PGC1α using actin as loading control and the densitometry analysis was performed (S5G Fig.). (E) The transcript levels of *Slc2a1* and *Slc2a4* were determined by qPCR at different points p.i. Values were normalized for uninfected BMMo cells. The transcription levels of *Slc2a2* and *Slc2a3* transcripts from WT infected BMMo were analysed (S5H Fig.). *Slc2a1* and *Slc2a4* transcripts from naïve and *L. infantum* infected splenic macrophages (S5I Fig.) and from irradiated *L. infantum* BMMo were analyzed (S5J Fig.). (F) At 18 hours p.i., the levels of *Slc2a1*, *Slc2a4* and *Ppargc1a* transcripts and (G and S6A Fig) glucose uptake (2-NBDG staining) were analyzed in WT and AMPK KO cells. (H) OCR and ECAR in uninfected and infected AMPK KO BMMo were measured under basal conditions. (I) The respective SRC were also determined. The bioenergetic profile was traced for OCR and ECAR in WT and AMPK KO cells (S6B Fig.). The ratio OCR/ECAR was also determined (S6C Fig.). Means ± SD are from three independent experiments. *p <0.05, **p <0.01, ***p <0.001.

Although both glycolysis and oxidative phosphorylation produce ATP, the energetic yield in the latter is higher. Therefore, we quantified the intracellular pools of ATP and AMP throughout the infection. Surprisingly, a significant increase in the AMP/ATP ratio at 10 and 14 hours post-infection was observed (Fig. 4A), in agreement with the variations in the total content of AMP and ATP (S5A-S5B Fig.). The variation on the nucleotide pool was dependent not only on the presence of live parasites, as no significant difference was observed between uninfected BMMo and those exposed to irradiated parasites (S5A-S5B Fig.), but also on the parasite dose (S5C Fig.). ATP reduction was exclusively observed in infected cells (S5D Fig.). Furthermore, amastigote-infected cells displayed a drop in the total ATP pool, although to a lower extent than macrophages infected with promastigotes (S5E Fig.).

A central metabolic sensor in response to nutrient and energetic restriction is AMPK (Austin and St-Pierre, 2012). Concomitantly with the increase on AMP/ATP ratio, AMPK is phosphorylated at Thr 172 (AMPK-P-Thr172) in cells infected with live promastigotes (Fig. 4B and 4C) or amastigotes (S5F Fig.). AMPK activation by phosphorylation is mediated by the upstream kinase LKB1 as Thr 172 is not phosphorylated in infected LKB1 KO BMMo (Fig. 4D and S5G Fig.). Aside PGC-1α, AMPK activation is also associated with induced expression of glucose transporters (GLUTs) encoded by *Slc2a* genes. *Slc2a4*, but not *Slc2a1* (Fig. 4E) or *Slc2a2–3* (S5H Fig.) transcripts were upregulated in cells infected with live parasites. An increase of *Slc2a4* and *Slc2a1* transcripts in splenic macrophages were also upregulated (S5I Fig.) which rules out a potential *in vitro* artifact. In opposition, irradiated parasites were unable to induce the expression of *Slc2a4* (S5J Fig.).

To gain insight on the role of the LKB1/AMPK axis, we infected BMMo devoid of AMPK or LKB1 with *L. infantum*. Our data demonstrated that in infected LKB1 KO BMMo, AMPK is not phosphorylated (Fig. 4D). Upon *Leishmania* infection of AMPK or LKB1 KO BMMo, both *Slc2a4* and *Ppargc1a* genes were not induced demonstrating that LKB1/AMPK signaling pathway controls their transcript levels in the context of *Leishmania* infection (Fig. 4F). As such, we next assessed whether AMPK impact on glucose uptake. In contrast to WT BMMo, infected AMPK KO BMMo failed to increment glucose uptake (Fig. 4G and S6A Fig.). Infected AMPK KO BMMo presented a higher fold increase in ECAR values than for WT BMMo when compared to uninfected cells at 18 hours p.i. (Fig. 4H and S6B Fig.). Importantly, no significant change on OCR measurement and SRC was noticed in infected vs. uninfected AMPK KO BMMo in comparison to WT cells (Fig. 4H-I). Consequently, infected AMPK KO BMMo presented a lower OCR/ECAR ratio compared to WT cells (S6C Fig.). Measurements at real time of the bioenergetic profile corroborated the higher aerobic glycolytic flux and unchanged respiratory capacity for infected AMPK KO BMMo (S6B Fig.). Altogether, our data demonstrated that *L. infantum* induces the activation of AMPK in macrophage-infected cells controlling glucose uptake and respiration.

### SIRT1 regulates AMPK activation in *L. infantum*-infected macrophages

**Fig 5:**
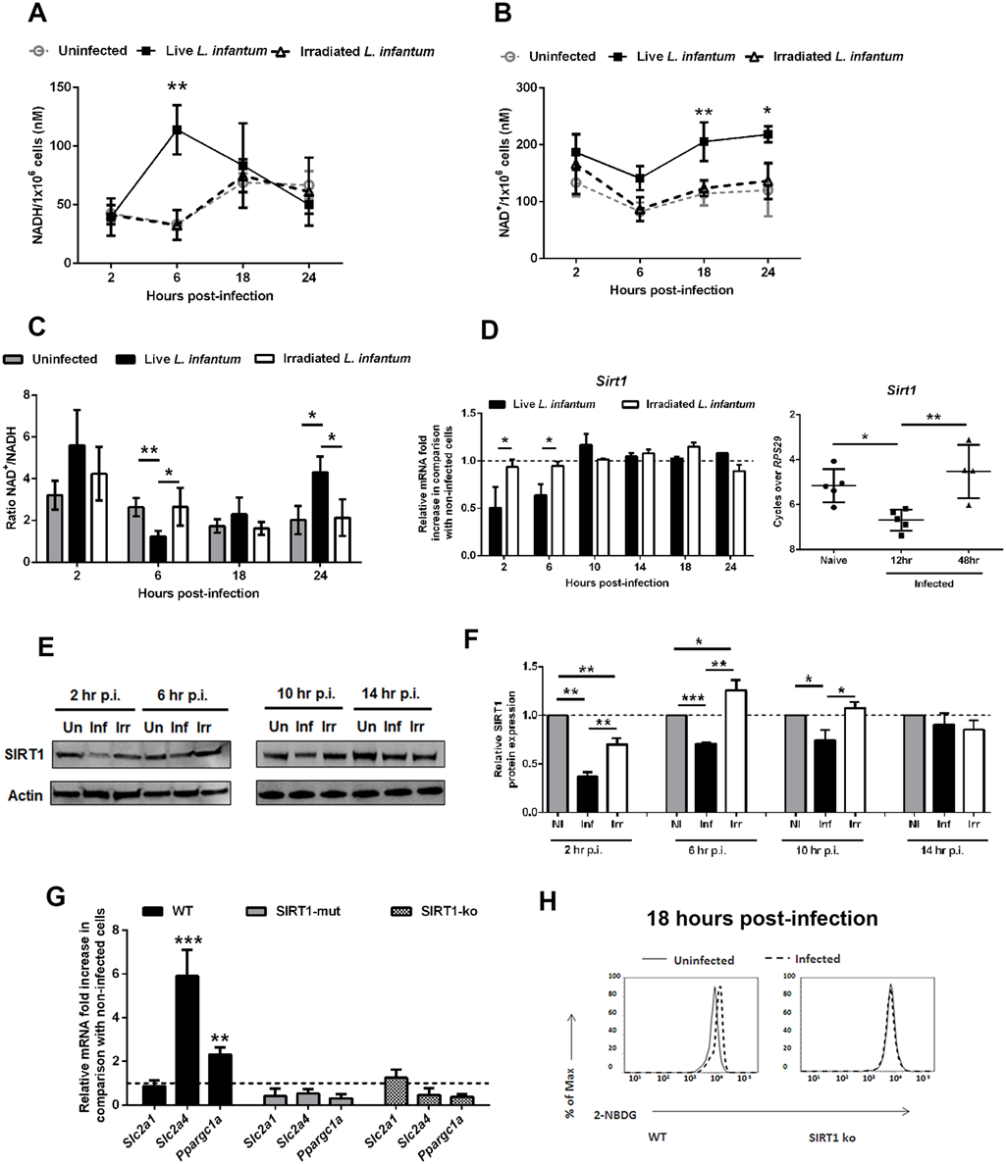
*L. infantum* modulates SIRT1 activity through redox state alteration. At different time points, the intracellular NADH (A), NAD^+^ levels (B) and NAD^+^/NADH ratio (C) were determined. *Sirt1* transcripts were analyzed by qPCR *in vitro* and *ex vivo* (D). Additionally the *in vitro* transcription of *Sirt3* and *Sirt6* were also determined (S7A Fig.). In distinct time points, whole cell extract immunoblots were probed with specific antibodies for SIRT1 and actin as loading control (E). Quantification of both proteins is expressed as folds in comparison to uninfected BMMo after normalization with actin (F). At 18 hours p.i., the levels of *Slc2a1*, *Slc2a4* and *Ppargc1a* transcripts (G and S7B Fig.) and glucose uptake (2-NBDG staining) (H) were measured in WT, Sirt1 knockout (KO) and Sirt1 catalytic mutant (SIRT1-mut) cells. Means ± SD are from four independent experiments. *p <0.05, **p <0.01, ***p <0.001.

A link between SIRT1 and AMPK has been previously described in several experimental models, allegedly acting as energetic sensors to sustain the metabolic homeostasis of the cell. Nevertheless, a clarification of the actual position of SIRT1 in relation to AMPK protein was needed. The aforementioned results prompted us to evaluate the levels of NAD^+^ and NADH nucleotides. BMMo infected with live parasites displayed a significant and transitory NADH increase at 6 hours p.i. (Fig. 5A). In contrast, NAD^+^ increased significantly in infected cells at 18 and 24 hrs (Fig. 5B), which reflected the higher NAD^+^/NADH ratio at these time points (Fig. 5C). Most importantly, a transient decrease in SIRT1 expression at the transcription and translational levels was observed both *in vitro* and *in vivo* infections (Fig. 5D-F). We also evaluated other members of the sirtuin family modulating metabolic pathways. However, we did not observe significant differences at the transcription levels of SIRT3 and SIRT6 during *L. infantum* infection (S7A Fig.). Due to the lack of specific chemical inhibitors for SIRT1, we used BMMo from mouse deficient or expressing an inactive form of SIRT1 [16]. Infected macrophages from these mouse strains failed to upregulate *Slc2a4* and *Ppargc1a* genes, resembling infected AMPK KO BMMo (Fig. 5G), and presented unchanged glucose uptake in comparison to uninfected cells (Fig. 5H and S7B Fig.).

**Fig 6:**
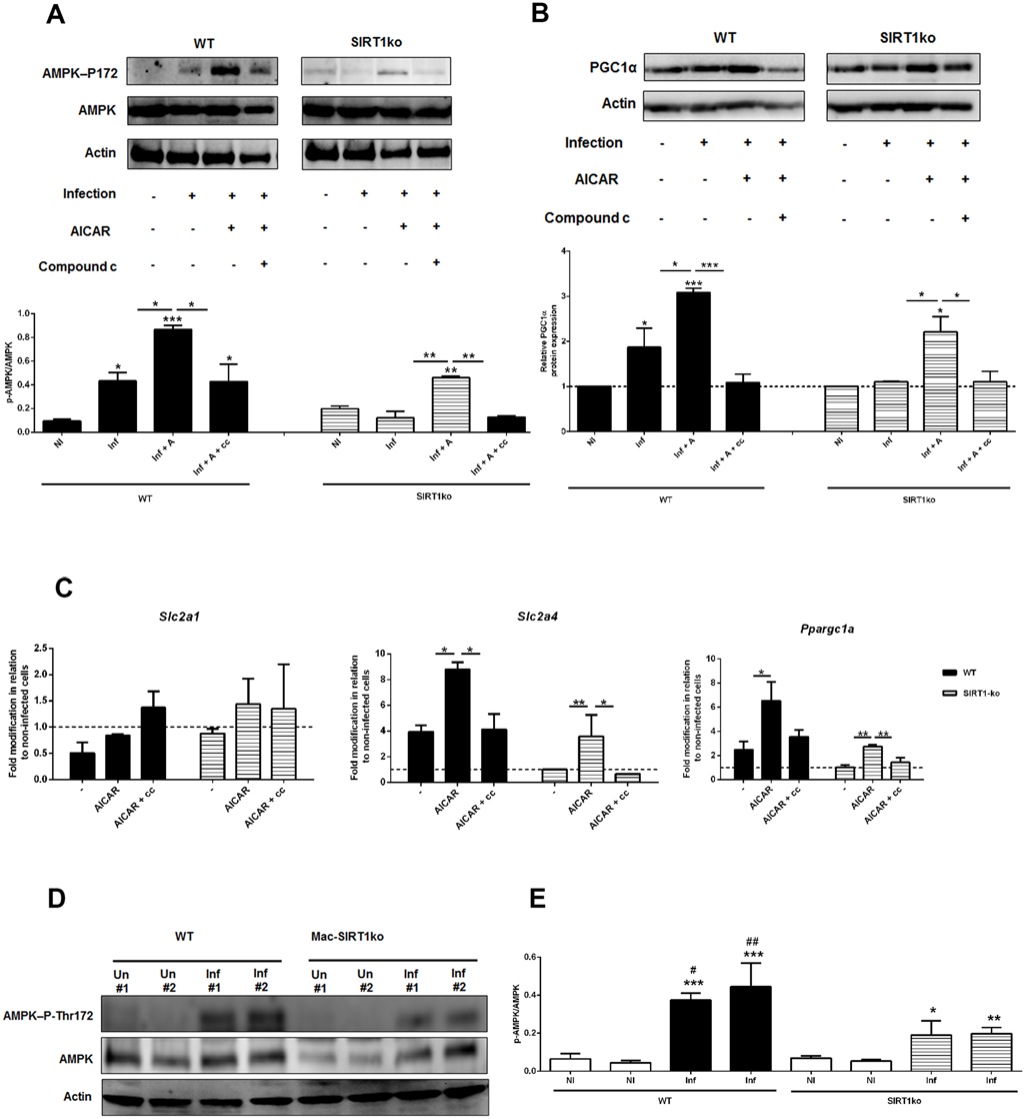
AMPK acts downstream SIRT1 in *Leishmania*-infected macrophages. Infected BMMo from WT and SIRT1 KO were treated with AICAR or simultaneously with AICAR and compound c. (A) At 14 hour post-infection the protein levels of AMPK-P-Thr172 and AMPK were determined. (B) At 18 hour post-infection the protein levels of PGC-1α were determined. Actin was used as loading control. Protein quantification is expressed as folds in comparison to uninfected BMMo after normalization with actin. (C) The levels of *Slc2a1*, *Slc2a4* and *Ppargc1a* transcripts from WT and SIRT1 KO infected and/or treated cells were analyzed by qPCR. (D) Whole cell extracts of macrophages recovered from the spleen of uninfected (Un) or 18-hour infected Mac-SIRT1 KO mice (Inf) were probed with specific antibodies for AMPK-P-Thr172 and AMPK. Actin was used as loading control. (E) Graphic represents the corresponding densitometry analysis. Means ± SD are from two representative mice from two independent experiments. *p <0.05, **p <0.01, ***p <0.001; Significant differences between WT and Mac-SIRT1KO splenic macrophages. ^#^p <0.05, ^##^p <0.01.

In order to dissect the cascade of events associated with SIRT1 and AMPK, we infected BMMo from WT and SIRT1 KO with *L. infantum*. The first evidence that AMPK is at the bottom of SIRT1 came from the absence of increase levels of AMPK-P-Thr172 in infected SIRT1 KO BMMo when compared to uninfected cells (Fig. 6A). Furthermore, the absence of SIRT1 activity influenced the up-regulation of PGC-1α protein expression (Fig. 6B). The addition of AICAR, an activator of AMPK, to infected WT or SIRT1 KO BMMo led to the activation of AMPK (Fig. 6A-B) and a significant increase in *Slc2a4* and *Ppargc1a* gene expression (Fig. 6C), which were abrogated when the AMPK inhibitor compound c was added simultaneously (Fig. 6A-C). Finally, the analysis of AMPK activation on splenic macrophages from uninfected and infected mice at 18 hours p.i. showed an increase of AMPK-P-Thr172 in splenic macrophages of infected WT mice (Fig. 6D-E). To note, AMPK-P-Thr172 was significantly reduced on splenic macrophages of SIRT1 KO infected mice (Fig. 6D-E). Overall, our results demonstrated the crucial role of SIRT1 in regulating AMPK activation in *L. infantum*-infected macrophages.

### Ablation of SIRT1 or AMPK induce parasite clearance *in vitro* and *in vivo*

**Fig 7:**
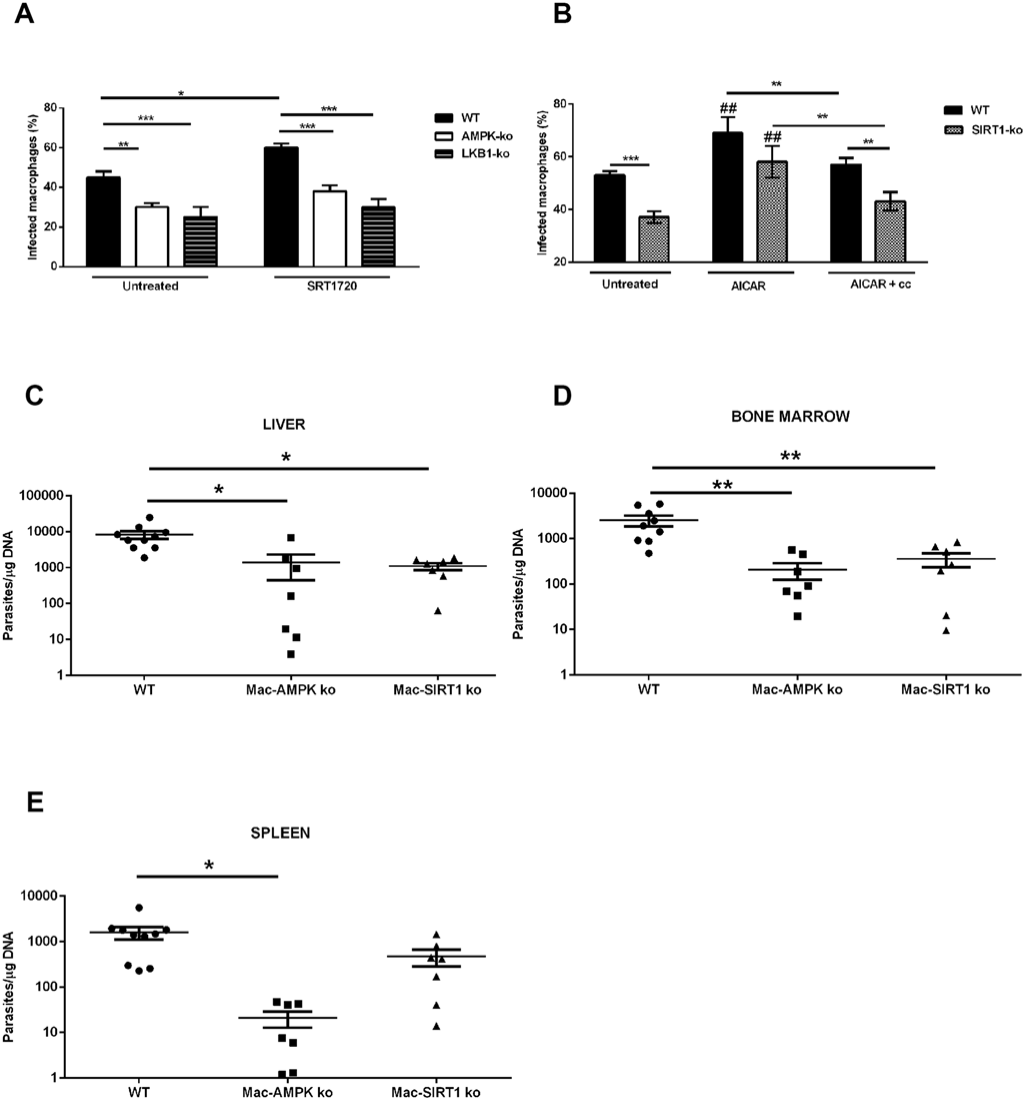
Inhibition of host AMPK contributes for *L. infantum* clearance. WT, AMPK or LKB1 knockout BMMo were infected with *L. infantum* promastigotes and treated or not with the SIRT1 activator SRT1720. The percentage of infected macrophages was quantified at 48 hours p.i. (A). The transcription levels of iNOS and Arg1 (S8A Fig.) and the respective ratio were determined (S8B Fig.). Percentage of infected BMMo at 48 hours p.i. from WT and SIRT1 KO treated with AICAR or AICAR plus compound c simultaneously (treatment was made at 6 hours p.i.) (B). The growth (S9A Fig.), viability (S9B Fig.) and infection of *L. infantum* promastigotes (S9C Fig.) were analyzed after AICAR, AICAR plus compound c or compound c treatment. Parasite load at day 10 of infection of the liver (C), bone marrow (D) and spleen (E) of *L. infantum* infected WT, Mac-AMPK KO and Mac-Sirt1 KO mice. Mean ± SD were obtained from seven to eight mice in each condition (see also S7–S8 Figs). *p <0.05, **p <0.01, ***p <0.001.

Our data demonstrated that *L. infantum* parasites exploit the SIRT1-LKB1-AMPK axis to shift macrophage metabolism towards mitochondrial oxidation. To unravel the role of SIRT1-LKB1-AMPK triad in the infection outcome, BMMo derived from AMPK, SIRT1 or LKB1 knockout mice were infected. All BMMo with a faulty active member of the triad were significantly more resistant to infection than the WT counterparts (Fig. 7A-B). Of note, the absence of AMPK leads to a shift on infected macrophages towards an inflammatory M1 profile, as represented by *iNOS/Arg1* ratio (S8A-B Fig.). As *Leishmania*-infected BMMo AMPK can be activated with AICAR in the absence of SIRT1 (Fig. 6A-B), the treatment with AICAR led to a significantly increase on infection levels, while the concomitant addition of compound c abrogated this infection rise to levels of untreated cells (Fig. 7B). To further validate this link, the percentage of infected BMMo from WT and SIRT1 KO cells treated with AICAR or either AICAR plus compound c was determined. The numbers of infected SIRT1 KO BMMo were significantly lower than their WT counterparts (Fig. 7B). Nevertheless, AMPK activation precluded this effect with infection rates similar to WT cells (Fig. 7B). Importantly, in the presence of compound c the infection returns to levels similar of untreated KO cells (Fig. 7B). Pre-treatment of *L. infantum* promastigotes with AICAR did not significantly altered the parasite growth curve, viability or the capacity to infect BMMo (S9A-S9C Fig.) discarding a direct effect on a potential *Leishmania* AMPK functional ortholog. An additional control was the observation of the lack of effect when infected AMPK or LKB1 KO BMMo were treated with the SIRT1 activator SRT1720 in comparison to WT cells (Fig. 7A).

Ultimately, a definitive demonstration of the biological role of AMPK and SIRT1 proteins was provided by infection of WT, myeloid restricted (Mac)-AMPK KO and Mac-SIRT1 KO mice with *L. infantum*, with evaluation of the parasite load in the spleen, liver and bone marrow 10 days post-infection. The absence of AMPK and SIRT1 led to a significantly reduction of the parasite load in all tested organs, except for the spleen of SIRT1 KO mice that showed a decreasing trend (Fig. 7C-E). Overall, the high correlation of AMPK and SIRT1 activities with parasite burden in *in vivo* infections underlines a new and high biologically relevant role for a metabolic control by the parasite.

## Discussion

**Fig 8:**
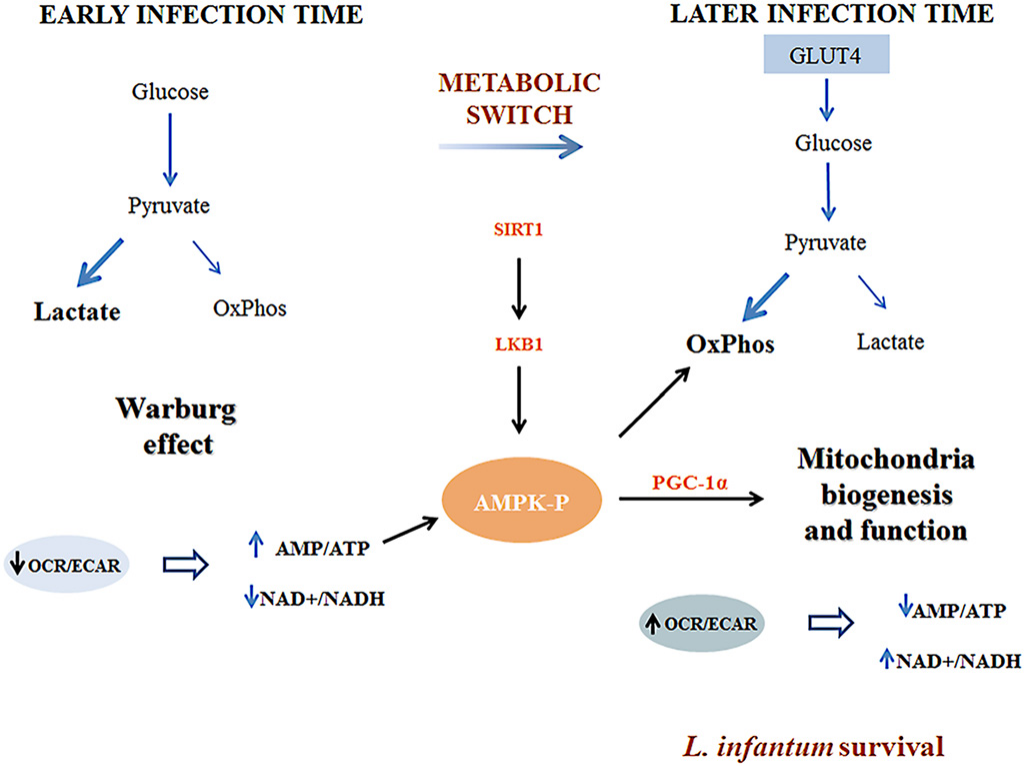
Representative scheme of the proposed model by which *Leishmania infantum* modulates host macrophage mitochondrial metabolism. At early stages of infection, *Leishmania infantum* modifies the transcriptional program of key glycolytic enzymes. This alteration drives the glucose towards the secretion of lactate decreasing at the same time mitochondrial glucose oxidation (OxPhos). As a result, a decrease on the OCR/ECAR ratio occurs and a deficit of the energetic and redox status are established in the infected cells. This leads to a metabolic switch where the high levels of AMP acting with the SIRT1-LKB1 axis led to the activation of AMPK. As a consequence, infected macrophages present increased expression of GLUT4 and PGC-1α as well as mitochondria biogenesis and respiration. This type of microenvironment has a profound impact on *Leishmaina* infection being more prone to the parasite survival inside the host.

Subversion of host cell energy metabolism by intracellular pathogens has been proposed to play a key role in microbial growth and persistence [6,17]. This topic is of extreme importance especially when lower eukaryotes as intracellular pathogens rely on a dramatic metabolic reprogramming to adapt to the challenges set by the new host nutritional status. Herein, we demonstrated that *L. infantum* infection of BMMo induced a switch towards mitochondrial oxidation phosphorylation favoring its own growth. We found that infection is associated with the activation of AMPK downstream to SIRT1 and LKB1. Indeed, BMMO defective in either SIRT1 or AMPK have reduced parasite growth. A representation of our proposed model is shown in Fig. 8.

Previous observations suggested that *Leishmania* can modulate metabolic pathways in their host cells to enhance parasite access to essential nutrients [17]. The transcriptomic signature of *L. major* infected macrophages identified anaerobic glycolysis as one of the major pathways regulated by the parasite early after infection [18]. Additionally, *L. major* was shown to inactivate mTORC1 decreasing macrophage global translation [19]. Although these studies suggest that subtle changes in the transcription and activities of host metabolic enzymes may have profound effects on the infection outcome, the mechanisms involved in the subversion of host metabolism and bioenergetic remodeling remain largely elusive.

The extracellular flux analysis with living cells allowed the bioenergetic evaluation of host macrophage upon challenge with *L. infantum*. We found that live parasites induced a rapid shift of macrophage metabolism towards aerobic glycolysis (Warburg effect) with a concomitant decrease of mitochondrial function. The observed increase on glycolytic pathways occurs specifically on infected cells although the transcription of *Pkm2* glycolytic enzyme is still induced on bystander macrophages (exposed to the parasite but not infected). *Pkm2* transcription has been related with macrophage [20,21] and NF-kB activation [22]. This phenomenon suggests that bystander macrophages could be activated in a similar fashion as it was demonstrated to occur with bystander dendritic cells [23,24] or even by infected macrophage secreted extracellular vesicles [25]. From the early energetic and metabolic settings responsible for a glycolytic environment resulted a nutrient and energetic deficit. Essential metabolites for the parasite including amino acids, purines, vitamins and haem have to be acquired from the host milieu [17]. This dependence on host purines could partially account for the ATP levels detected. Uninfected macrophages are known to rely on a glycolytic metabolism that could be explained by its presence in hypoxic inflamed tissues [26]. The yield of ATP produced by glycolysis is quite low. Macrophages, facing impaired respiration, respond by increasing their glycolytic rate in order to preserve the ATP pool and the glycolytic intermediates necessary to preserve mitochondrial membrane potential and viability [27]. Even though, the high dependency of infected macrophages on aerobic glycolysis with a concomitant impairment of mitochondria function in the early steps after infection reflects an even higher imbalance between these two pathways that may account the changes in ATP/AMP pool observed.

As a consequence of the higher intracellular AMP/ATP ratio, we detected the subsequent activation of AMP-activated protein kinase (AMPK), governing a known molecular stress response pathway regulating energy utilization and production [28]. AMPK activation stimulates a myriad of host catabolic processes to restore intracellular energy and nutrients, which can then be used to nourish the obligate intracellular pathogen. The activation of AMPK activity was followed by the raise of GLUT4 and PGC-1α, but not PGC-1β. PGC-1α induces a positive control over mitochondria biogenesis, increasing mitochondria functions and minimizing the buildup of its by-products, ensuring a global positive impact on oxidative metabolism [15]. In *L. infantum* infected cells the enhanced mtDNA/nDNA ratio supports an increase in abundance and functionality of mitochondria in the cell corroborating PGC-1α role.

SIRT1 and AMPK proteins share several downstream targets as PGC-1α [29], FOXO1 [30] and PPARα [31] as well as reciprocal regulation [32]. Some reports establish a functional link between these proteins demonstrating on the one hand a regulation of AMPK by SIRT1 through LKB1 deacetylation [33,34] and in contrast an activation of SIRT1 by AMPK dependent [35] or independent [28] induction of NAM phosphoribosyltransferase (Nampt) activity. Here, we demonstrated that in the absence of a catalytically active SIRT1, infected macrophages display a similar transcription and infection phenotype as AMPK KO cells. Our data suggests that both enzymes act synergistically to favor parasite growth. To elucidate their respective contribution, we activate SIRT1 in AMPK KO macrophages as well as the inverse strategy. Our data demonstrated that AMPK activation with AICAR is feasible in *Leishmania* infected SIRT1 KO/mutant cells, but not inversely, ranking SIRT1 upstream AMPK. A likely explanation is that SIRT1 exerts its activity over AMPK via the tumour supressor LKB1 kinase [33,34]. Indeed, LKB1 KO macrophages presented the same infection phenotype as SIRT1 KO BMMo. AMPK activation led to the reversion of *Ppargc1a* and *Slc2a4* transcription and consequently to increase infection burdens. AMPK plays a crucial role in the set-up of a mitochondria microenvironment, as suggested by the establishment of an aerobic glycolytic profile with a reduction on infection rate in its absence. The regulation of *Slc2a4* transcript by PGC-1α correlates AMPK activation with PGC-1α activity and place AMPK in the core of the transcriptional program of mitochondria function and biogenesis during *Leishmania* infection. Moreover, the absence of AMPK that impede the metabolic shift impacts the polarization profile of infected macrophages establishing a M1 inflammatory phenotype.

The change of metabolism during infection likely underlines the modification of cellular redox potential (NAD^+^/NADH ratio). The higher levels of NADH found in infected macrophages concomitantly to the establishment of aerobic glycolysis are potentially a consequence of reduced complex I activity, as suggested by the reduced *Ndufa9* transcript levels. NAD^+^ levels do not decrease significantly suggesting that NADH is at same extent being converted to NAD^+^ in the formation of lactate and reused by glycolysis to maintain the glycolytic flux. The decrease of NAD^+^/NADH ratio could be enough to reflect a reduction on SIRT1 energetic sensor activity that could actually contribute to the bioenergetic profile displayed by the infected cells in early time points. Of note, *Leishmania* parasites are NAD^+^ auxotroph using nicotinamide (Nam), among other precursors, for NAD^+^ synthesis [36]. This led us to hypothesize that the potential consumption of Nam by the parasite may lower its intracellular levels below the threshold necessary to induce a SIRT1 inhibitory effect [37]. Infections performed in mice deficient in the myeloid lineage for AMPK or SIRT1 demonstrated that the absence of each protein led to a significantly decrease on the liver, bone marrow and spleen parasite burden. The establishment of an *in vivo* microenvironment with an enhancement of mitochondria functionality in the presence of *L. infantum* is not only suggested by the described parasite burden but also by the phosphorylation of AMPK found in macrophages from the spleen of infected WT mice. Nevertheless, in an *in vivo* microenvironment, we cannot exclude other mechanisms/variables that could interfere with the host immune response against *L. infantum* infection. It is tempting to hypothesize that the manipulation of AMPK activation could be a common mechanism advantageously used by intracellular pathogens. In our model of infection, AMPK develops a crucial role for the persistence of the parasite inside the host. However, the acute silencing of AMPK catalytic or regulatory subunits favors intracellular *T. cruzi* growth [5]. Thus, even for parasites displaying a close phylogenetic relationship such as *Leishmania* and *T. cruzi* may exploit AMPK in a diametrically opposite manner.

Several *Leishmania* virulence factors, namely lipophosphoglycan and glycoprotein gp63, at the surface or released as exosomes [38,39], are known to modulate host signaling pathways interfering with the function of transcription factors and consequently altering host gene expression [40]. Herein, we found that energetic/metabolic alterations only occur in infected but not bystander cells. Moreover, irradiated parasites were unable to induce a similar phenotype, indicating that the observed variations are not due to the presence of the parasite itself but to the manipulation of host bioenergetics status by the parasite. Our results are also consistent with earlier observation that *Leishmania* has also the capacity to delay programmed cell death (PCD) induction in the infected macrophages by modulating apoptosis through mitochondrial permeabilization [41]. Thus, mitochondria represent for *Leishmania* one the main organelles targeted by the parasite not only to modulate cellular viability but also to control mitochondrial bioenergetics, as shown in the current work. Importantly, the increase of oxidative phosphorylation in infected macrophages is not exclusive for promastigote infection. Amastigotes also induced increased levels of *Pdk1* transcripts, however at lower extent, suggesting also an inhibition of the TCA cycle and mitochondrial respiration, which was supported by the decrease of ATP levels. Although we did not observed an early change in the levels of LDH activity/lactate secretion in, amastigote infection cells, the observed phenotype lead us to hypothesize that the possible inhibition of host PDH1 enzyme in our model of amastigote infection suggests a preventive role, developed by the parasite, in the production of reactive oxygen species (ROS) by host mitochondria as was already described under hypoxic conditions [42] and [43]. This is in agreement with the described capacity of *Leishmania* amastigotes to resist to the macrophage microbicidal activity in order to persist inside the host.

Similarly to the promastigote infection, we detected increased transcript levels of *Pparg1c* and mitochondrial biogenesis as well as increased levels of AMPK phosphorylation. Overall, our data places energetic deficit, AMPK activation and increased mitochondrial metabolism as a common mechanism induced by *L. infantum* irrespective of the infective parasite form.

In this study, we uncovered a subversion mechanism employed by *L. infantum* impacting host metabolic homeostasis. *L. infantum* interferes positively with AMPK pathway predisposing the host microenvironment for parasite growth. Our work underlines the potential of macrophage metabolism as a new therapeutic target to modulate leishmaniasis infection.

## Materials and Methods

### Animals and parasites

Balb/c, Mac-Sirt1 KO mice with 98% C57BL/6 background (myeloid cell-specific Sirt1 knockout mice), Mac-AMPKα1 KO, Mac-LKB1 KO and the respective littermate lox controls (Lysozyme-Cre^+/+^ Sirt1^flox/flox^) mice were maintained at the Instituto de Biologia Molecular e Celular (IBMC, Porto, Portugal) laboratory conditions, in sterile cabinets and allowed food and water *ad libitum*. All animals used in experiments were aged from six to twelve weeks. The enzyme-dead Sirt1-H355Y mice and the respective littermate lox controls were maintained at the animal facilities of Ottawa Hospital Research Institute. RS has an accreditation for animal research given from Portuguese Veterinary Direction (Ministerial Directive 1005/92). A cloned line of virulent *L. infantum* (MHOM/MA/67/ITMAP-263) were maintained by weekly subpassages at 26°C in RPMI 1640 medium (Lonza, Swtzerland) supplemented with 10% heat-inactivated Fetal Bovine Serum—FBS (Lonza, Switzerland), 2 mM L-glutamine, 100 U/ml penicillin, 100 mg/ml streptomycin and 20 mM HEPES buffer (BioWhittaker, Walkersville, MD). Only *L. infantum* promastigotes under four to ten passages were used in the experiments. Promastigote to amastigote differentiation was achieved by culturing 10^7^ stationary phase promastigotes/ml at 37°C in a cell free culture medium (MAA20). After a complete differentiation (3 days) the parasites were maintained by weekly subpassages.

### *In vitro* bone marrow macrophages differentiation and collection of peritoneal macrophages

Bone marrow precursors were recovered with DMEM medium after flushing femurs and tibias from the hind legs of Balb/c, AMPK KO mice, LKB1 KO, Sirt1 KO and Sirt1 mutant as described previously [44] with minor modifications. The hind legs of Sirt1 KO, Sirt1 mutant mice were kindly provided by Dr. Michael McBurney from Ottawa Hospital Research institute. The bone marrow cells obtained were suspended in complete macrophage medium (DMEM medium with glucose (4,5g/L) (Lonza, Switzerland) and HEPES buffer supplemented with 10% heat-inactivated Fetal Bovine Serum—FBS (Lonza, Switzerland), 2 mM L-glutamine, 100 U/ml penicillin and 100 mg/ml streptomycin (BioWhittaker, Walkersville, MD)) and 5% of L-929 cell conditioned medium (LCCM) was added. After 4h of incubation non-adherent cells were recovered and seeded at 5x10^5^cells/ml in 96, 24 and 6-well plates, in complete medium and 5% of LCCM, to continue bone marrow differentiation. Renewal of LCCM was made at day 4 of culture. Macrophages acquired a definitive differentiation status at day 7 of culture with a purity superior to 90%. For the recovery of peritoneal macrophages, Balb/c mice were injected intraperitoneally (i.p.) with ice-cold PBS. The inflated peritoneum was carefully shaken and the PBS solution removed. The macrophages purity was confirmed and then seeded in the same concentrations as BMMo. After an overnight incubation the cells were ready to use.

### *L. infantum* promastigotes staining

*L. infantum* promastigotes were used for CFSE (Invitrogen Molecular probes, Eugene, Oregon) and eFluor670 (eBioscience) labelling at a concentration of 6x10^7^ promastigotes/ml. Promastigotes were washed two times with PBS and labelled with 5 μM CFSE for 10 min or 1 μM eFluor670 for 5 min at 37°C followed by 5 min incubation at 4°C to stop the reaction. The parasites were then washed twice and suspended in RPMI supplemented medium before proceeding to infections as described before [45].

### *In vitro* macrophage infection

Seven-days differentiated BMMo were incubated with CFSE/eFluor670-*L*.*infantum* promastigotes at a 1:10 ratio. At same co-culture ratios, we performed experiments with irradiated (3000 Gy; Gammacell 1000 Elite) parasites and with unlabelled *L. infantum* amastigotes. After 4 hours of incubation, cells were washed to remove the non-internalized parasites, except for analyses made at 1 and 2 hours of infection. At defined time points the cells were recovered and the percentage of infected BMMo was determined by flow cytometry evaluation of CFSE^+^/eFluor670^+^ cells in a FACSCanto II cytometer (BD Biosciences) and analysed with FlowJo software (Tree Star, Ashland, OR). Growth curve and viability (1 μg/ml of 7-AAD) were assessed for both viable and irradiated promastigotes.

### Drug treatment

BMMo were treated at 6 hours post-infection with Aicar (440μM), Aicar + compound c (5μM), SRT1720 (1μM), Sirtinol (10μM), Resveratrol (1μM) and nicotinamide (1mM) that were then used for further analysis. At defined time points post-infection, BMMo were recovered and the percentage of infected cells was determined by flow cytometry evaluation of eFluor670^+^ or CFSE^+^ cells in a FACSCanto II cytometer (BD Biosciences) and analyzed with FlowJo software (Tree Star, Ashland, OR). *L. infantum* promastigotes were also treated during 18h with Aicar (440μM), Aicar + compound c (5μM) and compound c. The parasites were washed intensively to remove the excess of drug. Viability and growth curves were determined as well as BMMo infection rate. All the compounds used were obtained from Sigma-Aldrich (St. Louis, MO).

### Glucose uptake assay

Uninfected or eFluor-labelled *L. infantum* infected cells were incubated with 90 μΜ of 2-[N-(7-nitrobenz-2-oxa-1,3-diazol-4-yl)amino]-2-deoxy-D-glucose (2-NBDG) (Cayman Chemical, Michigan), a fluorescent analogue of glucose, in DMEM supplemented medium without glucose for 1 hour at 37°C. Cells were twice washed with cold PBS, harvested and stained with 1 μg/mL of *7-Aminoactinomycin D* (7-AAD) (Sigma, Saint Louis, Missouri). The 2–NBDG uptake was separately quantified on live eFluor^-^ and eFluor^+^ BMMo in a FACSCanto II cytometer and analyzed with FlowJo software.

### Cell sorting

*Bone marrow macrophages*: BMMo were infected with CFSE-labeled parasites and sorted according to their F4/80^+^ CFSE^+^ or F4/80^+^ CFSE^-^ for infected and bystander cells, respectively or F4/80^+^ staining in the case of non-infected cells.

*Splenic macrophages*: Mac-SIRT1 KO and littermate lox controls were infected intraperitoneally with 1 × 10^8^ CFSE-labeled *L. infantum* promastigotes. Naïve and infected mice were euthanized at 12, 18 or 48 hours post-infection and the spleens removed. Splenic T and B lymphocytes were depleted using the CD3ε and the CD19 microbeads coupled with LD columns (Miltenyi Biotec). The remaining cell suspension was labeled with anti-CD11b-PE, anti-Ly6C-PerCP/Cy5.5 and anti-Ly6G-Pacific Blue and sorted according the surface expression of CD11b^+^Ly6C^int/high^Ly6G^low^ and CFSE expression gated on infected (CFSE^+^CD11b^+^Ly6C^int/high^Ly6G^low^) or bystander (CFSE^-^CD11b^+^Ly6C^int/high^Ly6G^low^) splenic macrophages. For all the experiments, CD11b^+^Ly6C^int/high^Ly6G^low^ cells from the spleen of non-infected mice were sorted as a control. Sorting experiments were performed in a FACSAria I using FACSDiva software (BD Biosciences). The purity of the separation was always higher to 90% as confirmed by flow cytometry.

### *In vivo* experiments

WT, Mac-Sirt1 KO and Mac-AMPK KO mice were infected intraperitoneally with 1 × 10^8^ *L. infantum* promastigotes resuspended in sterile PBS. Ten days post-infection, the animals were euthanized and the DNA from spleen, liver and bone marrow extracted with DNAzol Reagent (Invitrogen, Barcelona, Spain). The parasite burden was evaluated as previously described [46].

### Quantitative PCR analysis

Total RNA was isolated from cells with TRIzol reagent (Invitrogen, Barcelona, Spain) or RNeasy micro kit (Qiagen), according to the manufacturer instructions. To determine mitochondrial DNA (mtDNA)/nuclear DNA (nDNA) ratios, mitochondrial and genomic DNA was extracted by QIAmp DNA micro kit (Qiagen). DNA from spleen, liver and bone marrow were extracted by DNazol according to the manufacture instructions. The RNA and DNA concentration was determined by OD260 measurement using a NanoDrop spectrophotometer (Thermo Scientific, Wilmington, DE). Total RNA (10–200ng) was reverse-transcribed using the iScript Select cDNA Synthesis Kit (BioRad, Hercules, CA, USA). Real-Time quantitative PCR (qRT-PCR) reactions were run in duplicate for each sample on a Bio-Rad My Cycler iQ5 (BioRad). The mitochondrial DNA (mtDNA)/nuclear DNA (nDNA) ratio was quantify by qPCR as previously described [47]. Primer sequences were obtained from Stabvida (Portugal) and thoroughly tested. The resulting RT product was expanded using the Syber Green Supermix (BioRad). The results were then normalized to the expression of a housekeeping gene *Rps29*. After amplification, a threshold was set for each gene and cycle threshold-values (Ct-values) were calculated for all samples. Gene expression changes were analysed using the built-in iQ5 Optical system software v2.1 (BioRad). The complete list of primers used is represented in the S1 Table.

### Metabolism assays

Adenine nucleotide concentrations were determined in cell extracts by an enzymatic method [48]. As alternative, the ATP levels in macrophages were measured at each time point of infection. To this end, cells were washed with PBS and suspended in Glo-lysis buffer (Promega, Madison, Wisconsin). After 15 min of incubation, supernatants were recovered after centrifugation and used to quantify ATP by a luciferin–luciferase method using an ATP determination Kit (Molecular probes, Eugene, OR) according to manufacturer instructions. NAD and NADH levels were determined using Fluorescent NAD/NADH detection kit (Cell Technollogy, Inc, Montain View, CA) using 2x10^5^cells following protocols from the manufacturer. For the determination of the bioenergetic profile of infected BMMo, oxygen consumption rate (OCR) and extracellular acidification rate (ECAR) were determined at 6 and 18 hours post-infection using an XF-24 Extracellular Flux Analyzer (Seahorse Bioscience). BMMo after 7 days of differentiation were seeded at 2x10^5^cells/well in 400μl of complete macrophage medium in XF-24 cell culture plates. After an overnight period the cells were incubated with irradiated or not *L. infantum* promastigotes at a 1:10 ratio. One hour before the defined times of infection, the cells were washed and the medium change to XF medium (unbuffered DMEM supplemented with 4.5g/L of glucose, 2% of FBS, 2 mM L-glutamine, 100 U/ml penicillin and 100 mg/ml streptomycin). The real time measurement of bioenergetic profile was obtained under basal conditions and in response to oligomycin (1μM), fluoro-carbonyl cyanide phenylhydrazone (FCCP—1μM), Rotenone (1μM) and Antimycin A (1μM). The non-mitochondrial respiration was obtained by subtracting the Rotenone/Antimycin A values. The spare respiratory capacity (SRC) was obtained by subtracting FCCP from basal OCR values, and the glycolytic capacity defined as the variation between oligomycin and basal ECAR values. The procedure used in the experiments was established according to Seahorse manufacturer instructions. Lactate dehydrogenase (LDH) and lactate were measured on an AutoAnalyzer (PRESTIGE 24i, PZ Cormay S.A.) using reagents from the same provider. LDH catalysis the reduction of pyruvate by NADH, according the following reaction: Pyruvate + NADH + H+ → L-lactate + NAD+. The rate of decrease in concentration of NADH was measured photometrically at 340 nm, and it is proportional to the catalytic concentration of LDH present in the sample accordingly to the method described by Elliot and Wilkinson. Lactate is oxidized by lactate oxidase to pyruvate and hydrogen peroxide, which, in presence of peroxidase (POD), reacts with Nethyl-N-(2-hydroxy-3-sulfopropyl)-3-methylaniline (TOOS) forming a red compound, which colour intensity measured a t 546 and 700 nm is proportional to the concentration of lactate in the examined sample.

### Immunoblotting

*In vitro* and *ex vivo* macrophages (approximately 1×10^6^) and *L*.*infantum* promastigotes (1×10^7^), were lysed in ice-cold lysis buffer containing 50 mM Tris, pH 7.4, 1% Triton X-100, 150 mM NaCl, 10% glycerol, 50 mM NaF, 5 mM sodium pyrophosphate, 1 mM Na_3_VO_4_, 25 mM sodium-β-glycerophosphate, 1 mM DTT, 0.5 mM PMSF, and protease inhibitors (Complete Protease Inhibitor Cocktail; Roche), for 30 min at 4°C. The lysates (twenty to fifty micrograms of protein) were subjected to SDS-PAGE electrophoresis and the proteins were transferred to mini nitrocellulose membranes (Biorad) by the Trans Blot Turbo Transfer System (Biorad). The membranes were then incubated with primary antibodies and with horseradish peroxidase-coupled secondary reagents (Jackson ImmunoResearch). The membranes development was made by Super Signal West Pico or West Dura chemiluminescence substrate (Thermo Scientific). Primary antibodies were directed against: total AMPKα (23A3), AMPKα phosphorylated at Thr172 (#2531; both from Cell Signaling), total SIRT1 (H-300) and total PGC1β (E-9; both from Santa Cruz), total PGC1α (4C1.3; Merck Millipore), and β-actin (C4; Antibodies-online).

### Statistical analysis

Statistical analyses were performed using the Student’s t test for paired observations or one-way ANOVA test with a Bonferroni multiple-comparison posttest for multiple group comparisons. Statistically significant values are as follows: *p < 0.05, **p < 0.01, ***p < 0.001.

### Ethics statement

The experimental animal procedures were approved by the local Animal Ethics Committee of Institute for Molecular and Cell Biology, University of Porto, Portugal and licensed by DGV (Director of Veterinary, Ministry of Agriculture, Rural Development and Fishing, Govt. of Portugal in December 29, 2010 with reference 25406. All animals were handled in accordance with the IBMC.INEB Animal Ethics Committee and the DGV General guidelines and the principles and guidelines established in the *European Convention for the Protection of Vertebrate Animals Used for Experimental and Other Scientific Purposes* (Council of Europe, ETS no. 123, 1991).

## Supporting Information

S1 Fig Bioenergetic profile of live *L. infantum* infected cells. (DOCX)

S2 Fig Profile of irradiated *L. infantum* promastigotes. (DOCX)

S3 Fig Transcriptional control of glycolytic enzymes during *L. infantum* infection. (DOCX)

S4 Fig Enhancement of mitochondrial function at later stages of *L. infantum* infection. (DOCX)

S5 Fig Energetic and glycolytic fluctuations during *L. infantum* infection. (DOCX)

S6 Fig Glucose uptake and bioenergetic profile of *L. infantum* AMPK KO infected BMMo. (DOCX)

S7 Fig Absence of transcriptional modifications on SIRT3 and SIRT6. (DOCX)

S8 Fig AICAR effects on infection are not associated to a concomitant inhibition of a potential *Leishmania* AMPK ortholog. (DOCX)

S9 Fig Absence of AMPK leads to a shift on macrophage polarization during *L. infantum* infection. (DOCX)

S1 Table Compiles the list of primers used for qPCR. (DOCX)
