# Supplementary material for: Leishmania infantum Modulates Host Macrophage Mitochondrial Metabolism by Hijacking the SIRT1-AMPK Axis
Source: PLoS Pathog. 2015 Mar 4;11(3):e1004684. doi: 10.1371/journal.ppat.1004684 (PMC4349736; doi:10.1371/journal.ppat.1004684)
Supplement: S1 Fig — (DOCX) [file ppat.1004684.s001.docx]

**
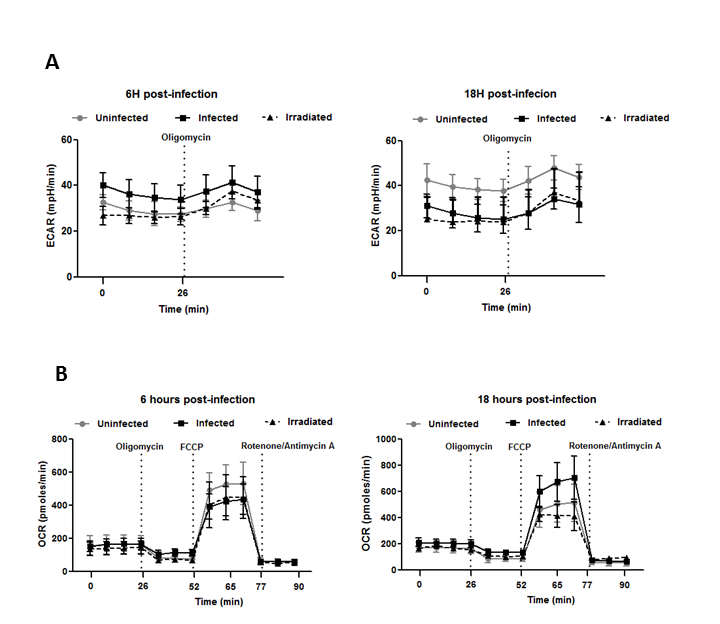
**

**S1 Fig. Bioenergetic profile of live *L. infantum* infected cells.**

BMMo were infected with live or irradiated *L. infantum* (1:10 ratio) for 6 and 18 hours. At each time point ECAR (A) and OCR (B) was determined in real time under basal conditions and in response to the indicated mitochondrial inhibitors. One representative experiment is shown from eight independent experiments. (*p <0.05).
