# Supplementary material for: Leishmania infantum Modulates Host Macrophage Mitochondrial Metabolism by Hijacking the SIRT1-AMPK Axis
Source: PLoS Pathog. 2015 Mar 4;11(3):e1004684. doi: 10.1371/journal.ppat.1004684 (PMC4349736; doi:10.1371/journal.ppat.1004684)
Supplement: S2 Fig — (DOCX) [file ppat.1004684.s002.docx]

**
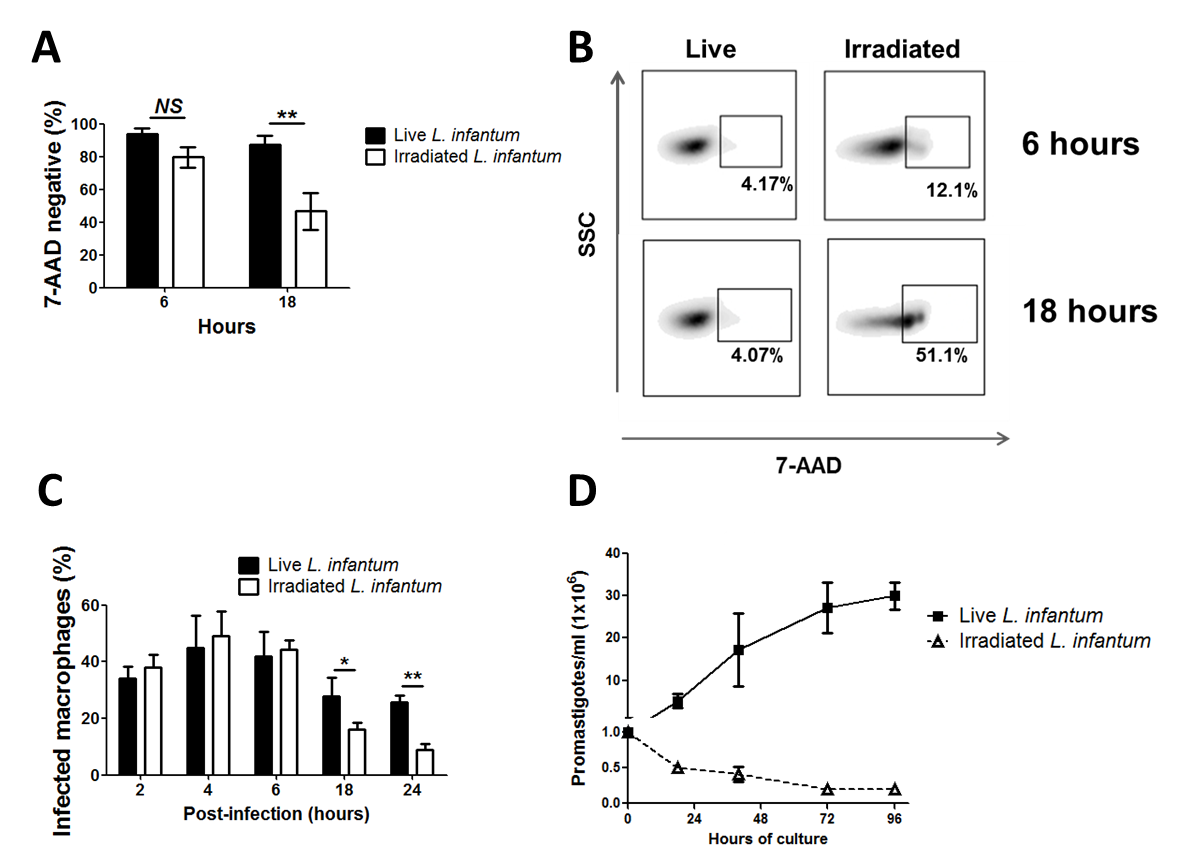
**

**S2 Fig. Profile of irradiated *L. infantum* promastigotes.**

*L. infantum* promastigotes were irradiated to 3000 Gy. (A) The viability of irradiated and non-irradiated parasites (live; corresponding to the same batch of parasites) was determined by 7-AAD staining in flow cytometry. (B) One representative example at 6 and 18 hours after the irradiation is depicted. BMMo were infected with live or irradiated *L. infantum* promastigotes (immediately after irradiation). (C) The percentage of infected macrophages was quantified until 24 hours pi. (D) Live and irradiated parasites were cultured at 1 x 10^6^ parasite/ml and followed during four days. The culture density was quantified at a daily basis. One representative experiment is shown from three independent experiments. (*NS* – non significant; *p <0.05; **p<0.01).
