# Supplementary material for: Leishmania infantum Modulates Host Macrophage Mitochondrial Metabolism by Hijacking the SIRT1-AMPK Axis
Source: PLoS Pathog. 2015 Mar 4;11(3):e1004684. doi: 10.1371/journal.ppat.1004684 (PMC4349736; doi:10.1371/journal.ppat.1004684)
Supplement: S3 Fig — (DOCX) [file ppat.1004684.s003.docx]

**A B**

**
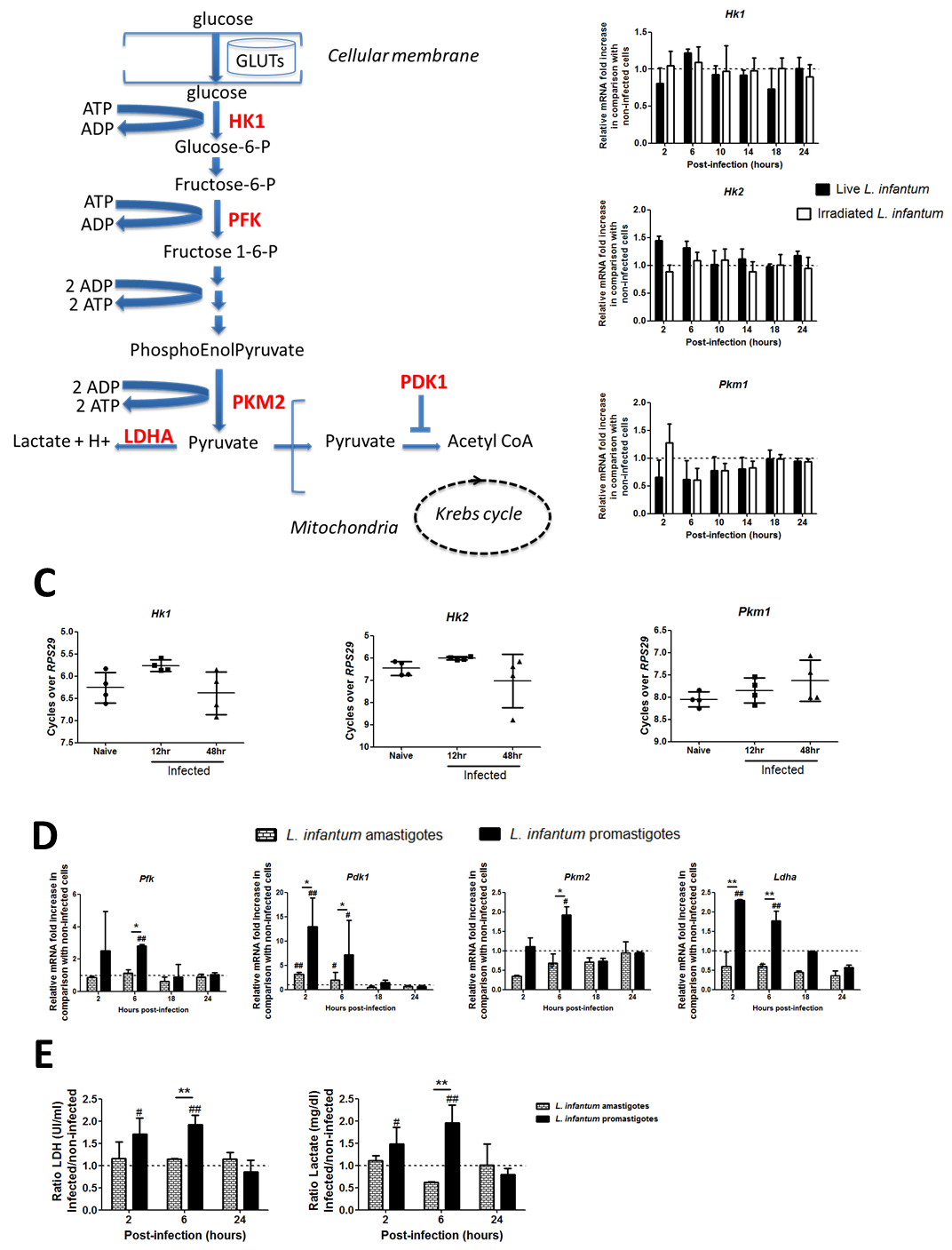
**

**S3 Fig. Transcriptional control of glycolytic enzymes during *L. infantum* infection.**

(A) Representative scheme of the glycolytic pathway highlighting the enzymes analyzed (in red). (B) BMMo were infected with live or irradiated *L. infantum* (1:10 ratio). The transcription levels of the glycolytic genes *Hk1, Hk2*, *Pkm1* were analyzed by qPCR in defined time points. Means ± SD are from three independent experiments. (C) *Hk1, Hk2* and *Pkm1* transcripts were determined in naïve and infected splenic macrophages analyzed *ex-vivo* at 12 and 48 hours post-infection. Means ± SD are from four individual animals. (D) BMMo were infected with live axenic *L. infantum* amastigotes or promastigotes (1:10 ratio). The transcription levels of the glycolytic genes *Pfk, Pdk1, Pkm2* and *Ldha* were analyzed by qPCR in defined time points. (E) LDH activity and lactate secretion were analyzed in the latter condition. Means ± SD are from two independent experiments. (*p <0.05, **p <0.001) Significant differences related to uninfected BMMo (^#^p <0.05, ^##^p <0.001).
