# Supplementary material for: Leishmania infantum Modulates Host Macrophage Mitochondrial Metabolism by Hijacking the SIRT1-AMPK Axis
Source: PLoS Pathog. 2015 Mar 4;11(3):e1004684. doi: 10.1371/journal.ppat.1004684 (PMC4349736; doi:10.1371/journal.ppat.1004684)
Supplement: S4 Fig — (DOCX) [file ppat.1004684.s004.docx]

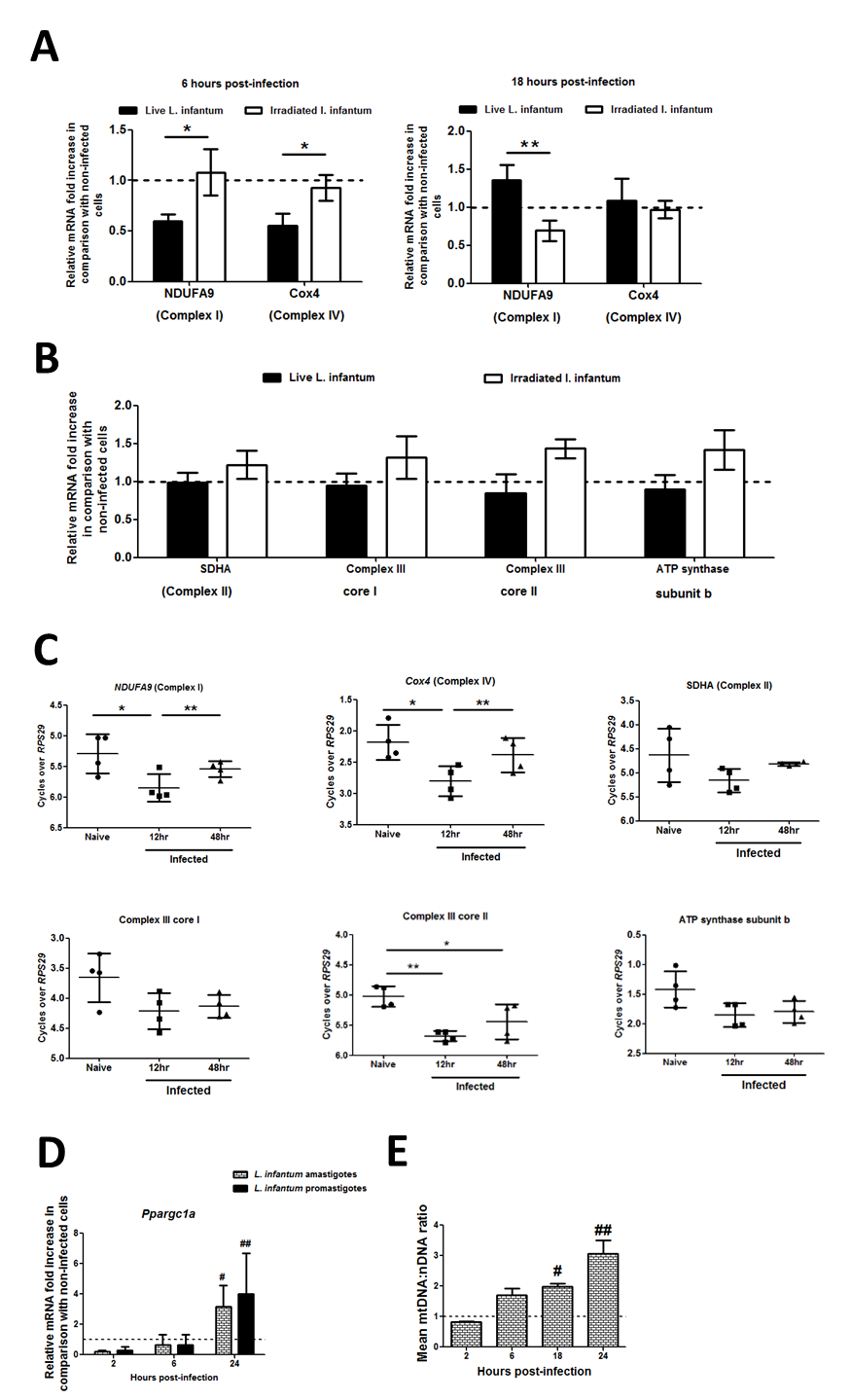


**S4 Fig.** **Enhancement of mitochondrial function at later stages of *L. infantum* promastigotes and amastigotes infection.**

BMMo were infected with live and irradiated *L. infantum* (1:10 ratio). At 6 and 18 hours post-infection the transcript levels of *Ndufa9* and *Cox4* (A) as well as *Sdha*, *Complex III core I*, *Complex III core II* and *ATP synthase subunit b* (all at 6 hours) were analysed by qPCR (B). A similar quantification was performed in naïve and infected macrophages recovered from the spleen of Balb/c mice after 12 and 48 hours of infection (C). BMMo were infected with live axenic *L. infantum* amastigotes or promastigotes (1:10 ratio). The *Ppargc1*a transcript (D) and the mtDNA/nDNA ratio were determined (E). Means ± SD are from three independent experiments. (*p <0.05, **p <0.001, ***p <0.0001).
