# Supplementary material for: Leishmania infantum Modulates Host Macrophage Mitochondrial Metabolism by Hijacking the SIRT1-AMPK Axis
Source: PLoS Pathog. 2015 Mar 4;11(3):e1004684. doi: 10.1371/journal.ppat.1004684 (PMC4349736; doi:10.1371/journal.ppat.1004684)
Supplement: S5 Fig — (DOCX) [file ppat.1004684.s005.docx]

**
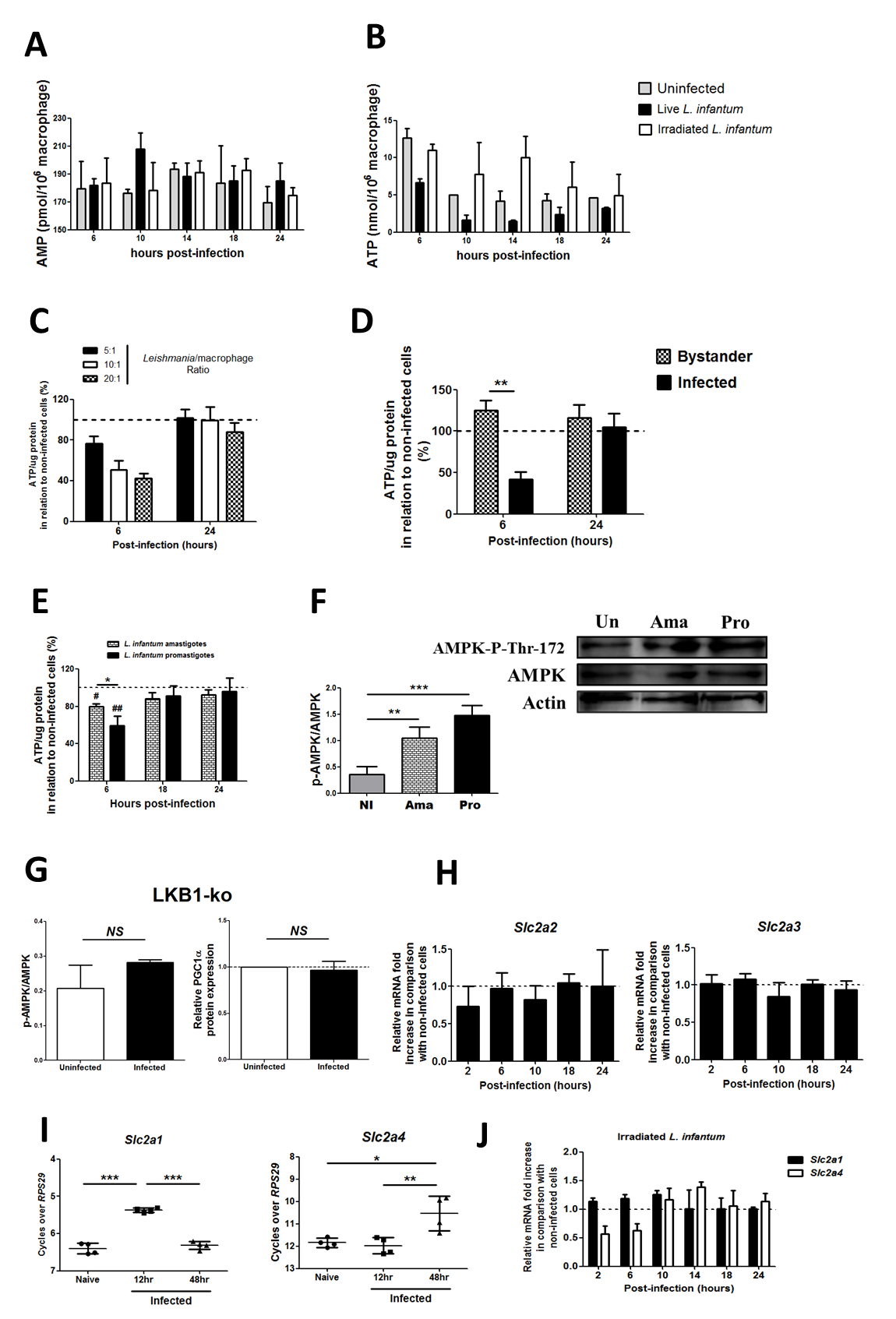
**

**S5 Fig.** **Energetic and glycolytic fluctuations during *L. infantum* infection.**

BMMo were infected with live and irradiated *L. infantum* (1:10 ratio). At different time points of infection AMP (A) and ATP (B) absolute levels were determined. Total ATP levels were determined in cells infected at different parasite doses (C) or in sorted infected and bystander cells (D). BMMo were infected with live axenic *L. infantum* amastigotes or promastigotes and the total levels of ATP were determined (E). At 10 hours post-infection, the levels of AMPK-P-Thr172 and AMPK were quantified. Graphic represents the corresponding densitometry analysis. Means ± SD are from two independent experiments (F). The levels of AMPK-P-Thr172, AMPK and PGC-1α were similarly quantified in uninfected and *L. infantum* infected BMMo recovered from LKB1-KO 14 hours p.i.. Graphic represents the corresponding densitometry analysis Means ± SD are from two independent experiments (G). The transcription levels of GLUTS were analyzed by qPCR. *Slc2a2* and *Slc2a3* transcripts from WT infected BMMo (H), *Slc2a1* and *Slc2a4* from naïve, *L .infantum* infected splenic macrophages (I) and irradiated *L. infantum* BMMo (J). Means ± SD are from three independent experiments. (*p <0.05, **p <0.001, ***p <0.0001). Significant differences related to uninfected BMMo (^#^p <0.05, ^##^p <0.001).
