# Supplementary material for: Leishmania infantum Modulates Host Macrophage Mitochondrial Metabolism by Hijacking the SIRT1-AMPK Axis
Source: PLoS Pathog. 2015 Mar 4;11(3):e1004684. doi: 10.1371/journal.ppat.1004684 (PMC4349736; doi:10.1371/journal.ppat.1004684)
Supplement: S6 Fig — (DOCX) [file ppat.1004684.s006.docx]

**
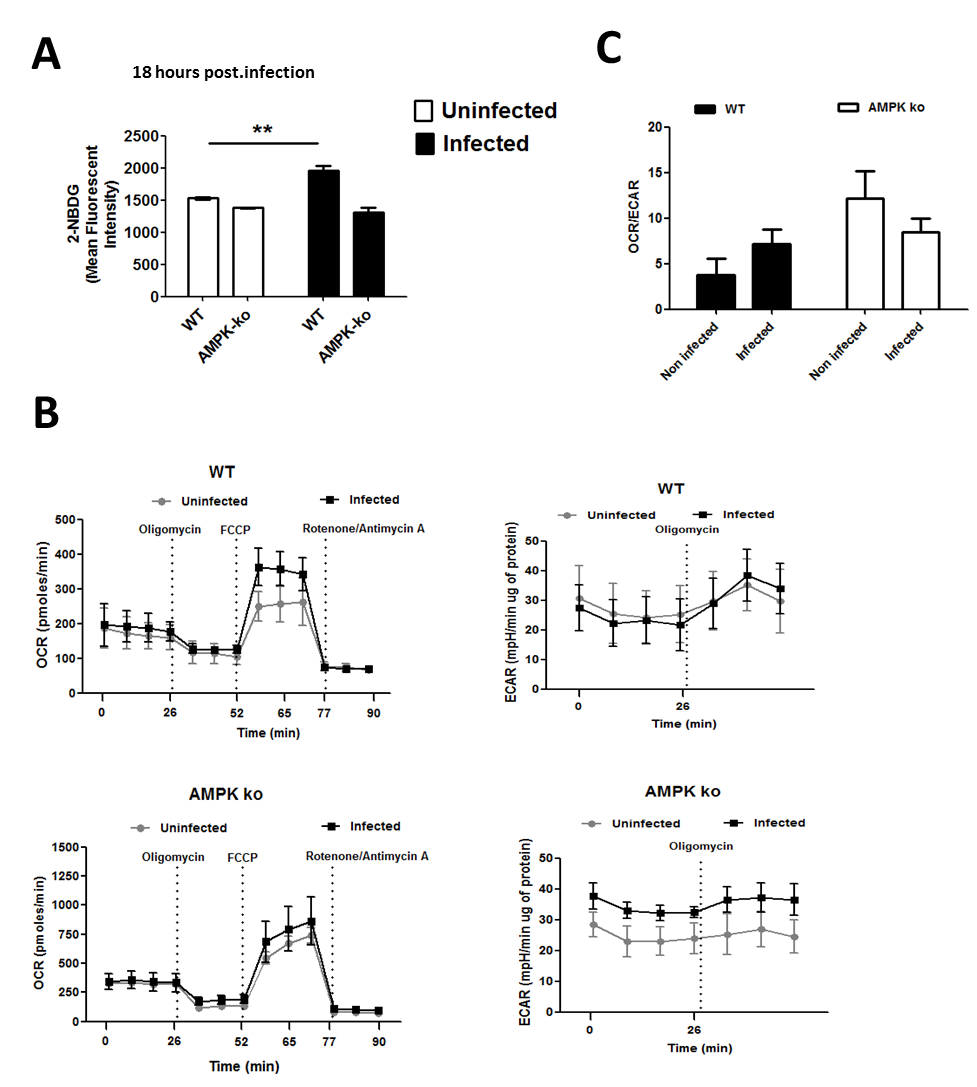
**

**S6 Fig. Glucose uptake and bioenergetic profile of *L. infantum* AMPK KO infected BMMo.**

BMMo from WT and AMPK KO cells were infected with *L. infantum* (1:10 ratio) for 18 hours. (A) The glucose uptake (2-NBDG staining) was measured as well as (B) the OCR and ECAR real-time values under the effect of distinct mitochondria inhibitors. (C) The OCR/ECAR ratio is shown. Means ± SD are from three independent experiments (**p <0.001).
