# Supplementary material for: Leishmania infantum Modulates Host Macrophage Mitochondrial Metabolism by Hijacking the SIRT1-AMPK Axis
Source: PLoS Pathog. 2015 Mar 4;11(3):e1004684. doi: 10.1371/journal.ppat.1004684 (PMC4349736; doi:10.1371/journal.ppat.1004684)
Supplement: S7 Fig — (DOCX) [file ppat.1004684.s007.docx]

**
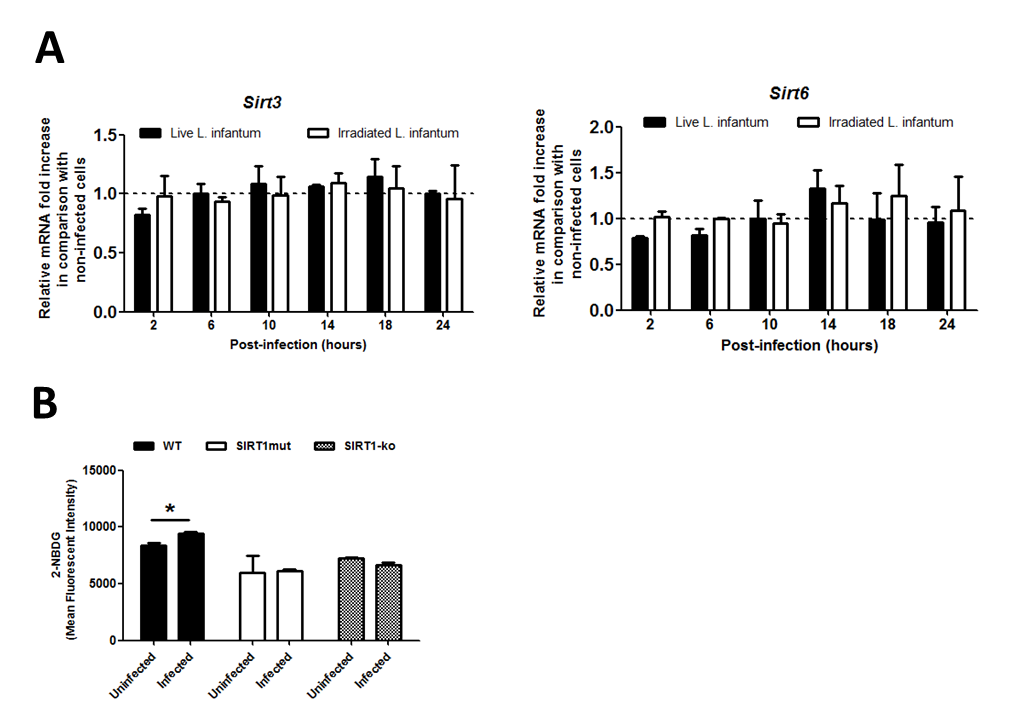
**

**S7 Fig. Absence of transcriptional modifications on SIRT3 and SIRT6.**

(A) BMMo were infected with live and irradiated *L. infantum* (1:10 ratio) at different time points of infection. The transcriptional profile of *Sirt3* and *Sirt6* transcripts were analysed at different time points. (B) BMMo from WT, SIRT1 mut and SIRT1 KO were infected with *L. infantum* (1:10 ratio) and the glucose uptake was measured by 2-NBDG staining. Means ± SD are from three independent experiments (*p <0.05).
