# Supplementary material for: Leishmania infantum Modulates Host Macrophage Mitochondrial Metabolism by Hijacking the SIRT1-AMPK Axis
Source: PLoS Pathog. 2015 Mar 4;11(3):e1004684. doi: 10.1371/journal.ppat.1004684 (PMC4349736; doi:10.1371/journal.ppat.1004684)
Supplement: S8 Fig — (DOCX) [file ppat.1004684.s008.docx]

**S8 Fig. Absence of AMPK leads to a shift on macrophage polarization during *L. infantum* infection.**

BMMo were infected with *L. infantum* (1:10 ratio). The transcriptional profile of *iNOS* and *Arg1* transcripts (A) and their ratio (B) were analyzed at 24 hours post-infection (*p <0.05 **p<0.01).
