# Supplementary material for: Leishmania infantum Modulates Host Macrophage Mitochondrial Metabolism by Hijacking the SIRT1-AMPK Axis
Source: PLoS Pathog. 2015 Mar 4;11(3):e1004684. doi: 10.1371/journal.ppat.1004684 (PMC4349736; doi:10.1371/journal.ppat.1004684)
Supplement: S9 Fig — (DOCX) [file ppat.1004684.s009.docx]

**
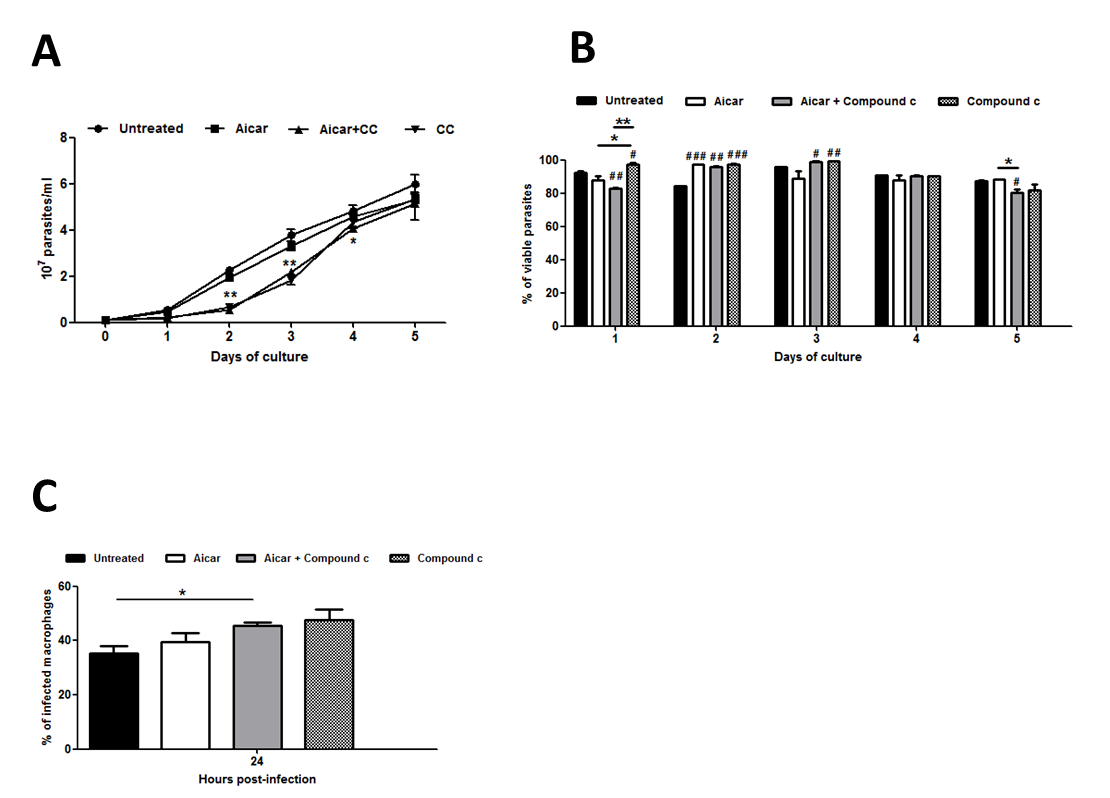
**

**S9 Fig. AICAR effects on infection are not associated to a concomitant inhibition of a potential *Leishmania* AMPK ortholog.**

*L. infantum* promastigotes were treated with AICAR, AICAR+compound c (cc) or compound c for 18 hours (A) Growth curve and (B) viability of untreated and treated parasites were analysed for the 5 days of culture. (C) BMMo were infected with parasites treated with AICAR, AICAR+compound c or compound c and the infection rate was determined. The percentage of infected macrophages was quantified at 24 hours pi. Means ± SD are from three independent experiments. (*p <0.05, **p <0.001, ***p <0.0001). Significant differences related to untreated parasites (^#^p <0.05, ^##^p <0.001, ^###^p <0.0001).
