# Supplementary material for: Leishmania infantum Modulates Host Macrophage Mitochondrial Metabolism by Hijacking the SIRT1-AMPK Axis
Source: PLoS Pathog. 2015 Mar 4;11(3):e1004684. doi: 10.1371/journal.ppat.1004684 (PMC4349736; doi:10.1371/journal.ppat.1004684)
Supplement: S1 Table — (DOCX) [file ppat.1004684.s010.docx]

S1 Table. List of primers used for qPCR

| **Genes** | **Forward Sequence** | **Reverse Sequence** | **Accession number** |
| --- | --- | --- | --- |
| ***Pfk*** | **GGAGGCGAGAACATCAAGCC** | **CGGCCTTCCCTCGTAGTGA** | **NM_008826** |
| ***Pdk1*** | **GGACTTCGGGTCAGTGAATGC** | **TCCTGAGAAGATTGTCGGGGA** | **NM_172665** |
| ***Pkm2*** | **TTGCAGCTATTCGAGGAACTCCG** | **CACGATAATGGCCCCACTGC** | **NM_001253883** |
| ***Ldha*** | **TGTCTCCAGCAAAGACTACTGT** | **GACTGTACTTGACAATGTTGGGA** | **NM_001136069** |
| ***Ppargc1a*** | **AGCCGTGACCACTGACAACGAG** | **GCTGCATGGTTCTGAGTGCTAAG** | **NM_008904** |
| ***Ppargc1b*** | **TGCCACAACCCAACCAGTCTCA** | **AGCAGTCTCCAGCAGCCCAAAG** | **NM_133249** |
| ***Slc2a1*** | **CCAGCTGGGAATCGTCGTT** | **AAGTCTGCATTGCCCATGAT** | **NM_011400** |
| ***Slc2a4*** | **ACATACCTGACAGGGCAAGG** | **CGCCCTTAGTTGGTCAGAAG** | **NM_009204** |
| ***Sirt1*** | **AGAACCACCAAAGCGGAAA** | **TCCCACAGGAGACAGAAACC** | **NM_001159589** |
| ***Sirt3*** | **TGCTACTCATTCTTGGGACC** | **CACCAGCCTTTCCACACC** | **NM_001127351** |
| ***Sirt6*** | **CATGGGCTTCCTCAGCTTC** | **AACGAGTCCTCCCAGTCCA** | **NM_181586** |
| ***HkI*** | **CGGAATGGGGAGCCTTTGG** | **GCCTTCCTTATCCGTTTCAATGG** | **NM_001146100** |
| ***HkII*** | **TGATCGCCTGCTTATTCACGG** | **AACCGCCTAGAAATCTCCAGA** | **NM_013820** |
| ***Pkm1*** | **TTGTGCGAGCCTCCAGTC** | **ACTCCGTGAGAACTATCAAAGC** | **NM_001253883** |
| ***Ndufa9*** | **TGGGAGAGAAGGAAGTGAGAAG** | **CACAGCAAGAAACCAACGATAA** | **NM_025358** |
| ***Cox4*** | **TGGCAAGAGAGCCATTTCTACT** | **GTGGGGAAAGCATAGTCTTCAC** | **NM_009941** |
| ***Sdha*** | **AATTAAGGCAAATGCTGGAGAA** | **TGCATGTTTAGGCGTAGTTCTG** | **NM_025333** |
| ***Complex III core I*** | **GGCTACTTTTTGGAACATCTGG** | **GCCCCAATACTCTCTACCTCCT** | **NM_025407** |
| ***Complex III core II*** | **AGAGAGCAGAATGGAGACAACC** | **AGCACCAAGAAGATGCTGAAGT** | **NM_025899** |
| ***Atp synthase subunit b*** | **GTGGTTCGTTCATCTGTCCATA** | **GTATTTGCTGGTGTTGGTGAGA** | **NM_009725** |
| ***nuclDNA (Pecam 1)*** | **ATGGAAAGCCTGCCATCATG** | **TCCTTGTTGTTCAGCATCAC** | **NC_000077.6** |
| ***mtDNA (NADH dehydrogenase 1, mitochondrial)*** | **CCTATCACCCTTGCCATCAT** | **GAGGCTGTTGCTTGTGTGAC** | **NC_005089.1** |
| ***RPS29*** | **CACCCAGCAGACAGACAAACTG** | **GCACTCATCGTAGCGTTCCA** | **NM_009093** |
